# QSAR Models for Climate Impact Assessment: Born to Be Sustainable

**DOI:** 10.3390/toxics14080667

**Published:** 2026-07-28

**Authors:** Arianna Sgariboldi, Nicola Chirico, Ester Papa

**Affiliations:** 1 QSAR Research Unit in Environmental Chemistry and Ecotoxicology, Department of Theoretical and Applied Sciences, University of Insubria, via J.H. Dunant 3, 21100 Varese, Italy; asgariboldi@uninsubria.it (A.S.); nicola.chirico@uninsubria.it (N.C.); 2 Department of Science and High Technology, University of Insubria, via Valleggio 11, 22100 Como, Italy

**Keywords:** Global Warming Potential, Radiative Efficiency, in silico models, QSAR, LCIA, climate indicators

## Abstract

The Global Warming Potential (GWP) is among the metrics used as midpoints in the Life Cycle Impact Assessment (LCIA) phase of the Life Cycle Assessment. However, GWP values are not always available. In recent years, predictive modelling approaches have been increasingly developed to address existing data gaps. In this study, new Quantitative Structure–Activity Relationship (QSAR) models were developed to support climate impact assessment by predicting GWP and Radiative Efficiency of chemicals from the molecular structure. These models, which are built using Multiple Linear Regression, are designed to comply with the OECD (Organization for Economic Co-operation and Development) principles for QSAR use for regulatory purposes: they are transparent, validated, characterized by a quantitative domain of applicability and based on interpretable molecular descriptors. These QSARs are characterized by lower complexity, in terms of the number and type of descriptors, and easier applicability than other models with similar performance available in the literature for the same endpoints. A dedicated case study shows how the new QSARs can be applied to screen a large dataset including more than 13,000 possible alternatives to harmful chemicals. These results show that the new models may be suggested as valuable support in LCIA procedure and for the screening of environmentally preferable and safer alternatives.

## 1. Introduction

The sustainability of chemicals has become a global priority within environmental and industrial policy frameworks. The United Nations Member States adopted the 2030 Agenda for Sustainable Development in 2015, which identified 17 Sustainable Development Goals (SDGs), among such as Goal 3 (good health and well-being), Goal 6 (clean water and sanitation), Goal 9 (industry, innovation and infrastructure), and Goal 13 (climate action) explicitly highlighting the need for safer management and sustainable use of chemicals [1,2]. In Europe, these principles were operationalized in 2019 through the adoption of the European Green Deal, which aims to achieve climate neutrality by 2050 [3]. Building on this strategy, the European Commission (EC) published the Chemicals Strategy for Sustainability (CSS) in 2020, aiming for identifying and restricting hazardous substances and, where possible, substituting them with safer alternatives [4]. In December 2022, the EC introduced the Safe and Sustainable by Design (SSbD) framework, as a voluntary approach to guide the innovation process for chemicals and materials. The aim of this strategy can be summarized as steering innovation towards clean and sustainable industries, reducing or replacing substances of concern beyond regulatory requirements, and minimizing impacts on health, climate, and the environment across the entire life cycle of chemicals and materials [5]. One of the key stages integrated into the SSbD framework is Life Cycle Assessment (LCA) [5,6,7,8], an ISO-standardized methodology for quantifying the potential environmental and human health impact associated with materials or services across their entire life cycle [9,10]. It is one of the methods developed to measure sustainable development, carry out environmental assessments, and evaluate sustainable chemical alternatives [11,12,13].

According to ISO 14044, LCA consist of four main phases: goal and scope definition, Life Cycle Inventory (LCI) analysis, Life Cycle Impact Assessment (LCIA); and interpretation of the results [9,10,14,15]. In the LCIA phase impacts are evaluated across different areas of protection, i.e., environment, human health, and resource availability, using specific impact indicators and midpoints [10].

Within the LCIA, Global Warming Potential (GWP) and Ozone Depletion Potential (ODP) are midpoints used to quantify contributions to the impact categories of climate change and ozone depletion, within the area of environmental protection [14].

GWP is one of the most widely used indicators for comparing potentially sustainable chemical alternatives. GWP was introduced by the Intergovernmental Panel on Climate Chance (IPCC) as a metric to assess the relative impact of greenhouse gases on global climate change [16]. Conventionally, GWP values are expressed relative to carbon dioxide (CO_2_) as the reference gas [16,17,18,19]. The GWP of a molecule is defined as “*the time-integrated global mean Radiative Forcing resulting from the pulse emission of 1 kg of the substance relative to that of 1 kg of reference gas*” (i.e., CO_2_) [16]. The calculation of GWP requires two key parameters. The first one is the Radiative Forcing (RF) which is defined as “*measure of the influence a factor has in altering the balance of incoming and outgoing energy in the Earth–atmosphere system and is an index of the importance of the factor as a potential climate change mechanism. It is expressed in Watts per square meter (Wm^−2^)*” [16]. When RF is applied to greenhouse gases, *the change normally considered is a 1 ppb increase in the concentration of the gas in the troposphere and is termed Radiative Efficiency (RE) units of W m^−2^ ppb^−1^* [20,21]. The second one is the Atmospheric Lifetime, defined as “*the time a gas remains in the atmosphere before it is completely degraded*” [22]. Although Atmospheric Lifetime significantly influences GWP values, Radiative Efficiency is often the dominant factor driving large variations among molecular species [23]. GWP values are periodically updated by IPCC and World Meteorological Organization (WMO) and reported for different time horizons (20, 100, and 500 years).

The calculation or availability of these indicators often relies on complex atmospheric modelling or experimental datasets that remain unavailable for many emerging, newly designed or insufficiently studied substances [24,25,26,27]. To overcome this limitation, it is recommended to use advanced predictive approaches, including machine learning techniques and computational chemical design [17,27,28,29,30]. These approaches are already used in different publications to predict data for LCIA or/and LCI [17,24,27,28,29,31,32,33,34,35,36,37,38,39,40,41].

A comparative overview of selected representative studies is provided in Table S1 (Supplementary Materials File S2) with related references.

The aim of this work is to develop QSAR models to predict GWP in a 100-year time horizon (the horizon commonly used in LCA evaluations) directly from the molecular structure of chemicals. In addition, a QSAR model to predict RE (which is an intermediate parameter for GWP calculation) is proposed to support the evaluation of potentially sustainable alternatives for which experimental data are lacking. Differently from most of the existing computational works published in this area and listed in Table S1, well-established principles and methodological good practices from the regulatory use of QSAR under the REACH regulation [42,43,44,45,46] are applied here to reduce uncertainty in prediction and increase the reliability of the assessment. It is worth pointing out that among the existing approaches based on QSAR-type models, only Song and colleagues [39] introduced the concept of an applicability domain within LCA. Furthermore, they highlighted the need for rigorous cross-validation procedures to avoid bias in training data selection and for the explicit incorporation of prediction uncertainty to enable the reliable use of predictive models in impact assessment studies. Notwithstanding these recommendations which align with the Organization for Economic Co-operation and Development (OECD) guidance for regulatory use of QSARs, models by Song and colleagues [39] built using Artificial Neural Networks (ANN) had poor fitting and predictive ability and could be improved.

Building upon these considerations, as was mentioned above, the present work adopts model development practices already established for regulatory acceptance of QSAR models [45], while paying particular attention to models’ fitting and predictive quality. By transferring these procedures into the LCA framework, we aim to enhance transparency and reproducibility to generate reliable predictions. For this reason, models should be simple, easily implemented for application, and interpretable, while predictions should be reliable. These are essential characteristics for regulatory acceptance. This is particularly relevant as complex machine learning algorithms (e.g., neural networks or random forest) may achieve high fitting and predictive accuracy, while presenting limited interpretability, which makes these models not always adequate for use in regulatory or sustainability assessment contexts [47].

## 2. Materials and Methods

### 2.1. Data Curation

**Figure 1:**
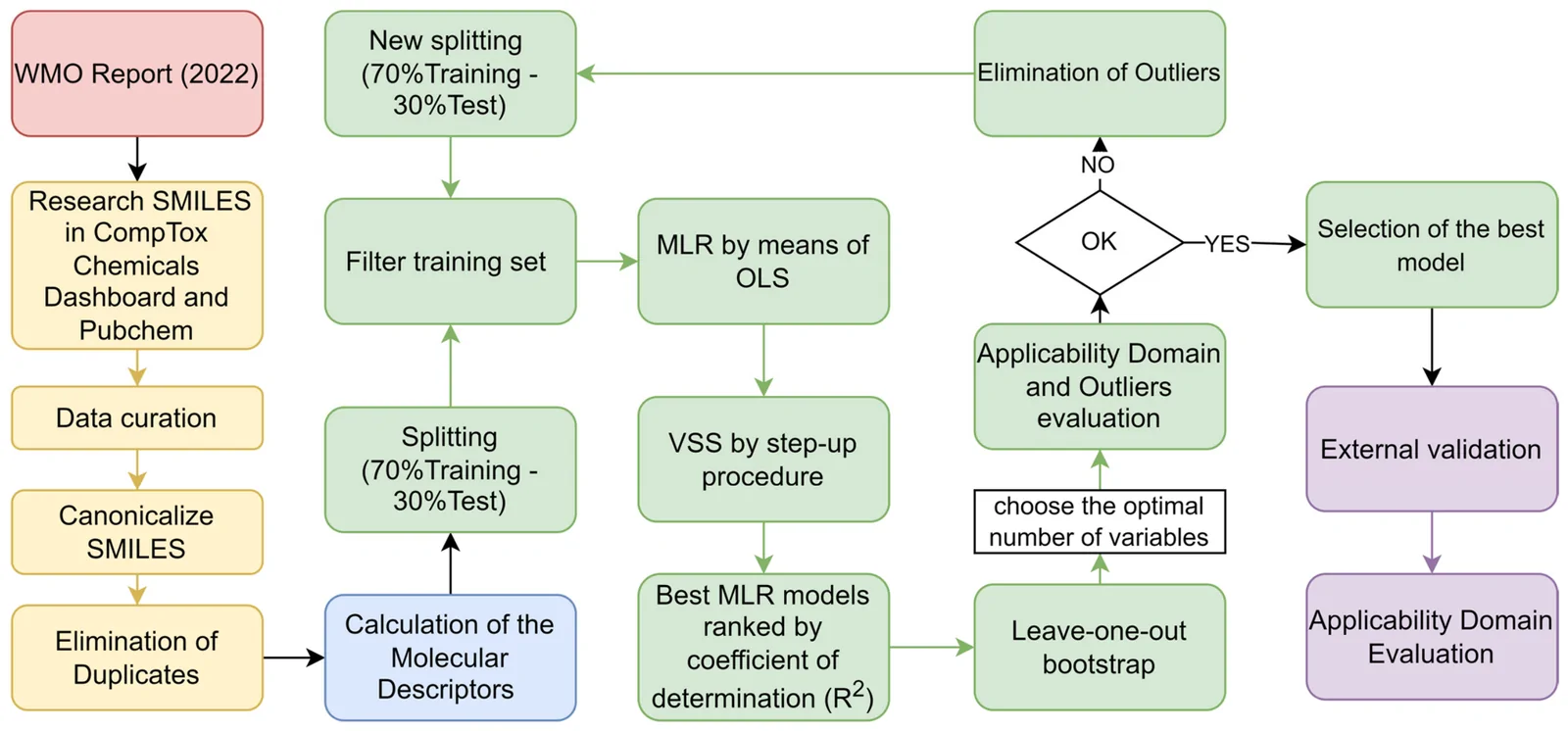
QSAR model development procedure used in this study.

Figure 1 outlines the procedure adopted in this study. A dataset was developed from the latest Scientific Assessment of Ozone Depletion report published by WMO in 2022 [22], which included the following properties for 782 substances: Atmospheric abundance, Total Lifetime, Tropospheric (OH Reactive Loss) Lifetime, Stratospheric Lifetime, ODP, RE (well mixed), Recommended Adjusted Effective Radiative Efficiency, GWP at 20, 100 and 500 year time horizons and Global Temperature chance Potential (GTP) at 50 and 100 year time horizons. GWP at 100-year time horizon and RE were chosen in this work as primary for the development of QSARs. In addition, the information on chemical classes available for each substance was used to support the interpretation of QSAR predictions. The raw dataset containing only the endpoints of interest for this work is provided in Supplementary Materials File S1 (Sheet 00).

### 2.2. Calculation of Molecular Descriptors and Dataset Splitting for External Validation

In order to calculate the independent variables to build the QSAR models, i.e., the molecular descriptors, the original dataset was curated by searching for the Simplified Molecular Input Line Entry System (SMILES) notations of chemicals through the US EPA CompTox Chemicals Dashboard [48] and the Pubchem database [49]. The above databases were used between the months of June and July 2025. The research was performed using both CASRN and chemical name, both provided in the original dataset in the WMO report [22].

SMILES notations were canonicalized, after the elimination of salts, mixtures and metalorganic compounds using Open Babel software v.2.4.1. [50] before calculating the molecular descriptors, to ensure consistency in the structural representation. The endpoint of the model was the logarithm (based 10) of the properties (i.e., GWP_100yr_ and RE).

Values expressed as > or < or ~ were not included in the dataset as well as those equal to zero.

The modelled datasets are reported in Supplementary Material File S1 (Sheet 02, Sheet 04) for each modelled endpoint. We used Mordred version 1.2.0 [51] to calculate one-dimensional and two-dimensional theoretical molecular descriptor and CDK version 2.9 (https://cdk.github.io/ (accessed on 20 October 2025)) to calculate the fingerprints.

The final datasets were split into a training set for QSAR development and a test set for external validation. The split was performed by keeping 70% of the information in the training set and 30% in the test set, following a scheme 2:1, after ordering the molecules by increasing values of the response and keeping the largest and the smallest values in the training set. The test set was defined prior to model development and was completely excluded from descriptor selection, variable-selection and applicability-domain analysis and it was used only to test the model’s external predictivity after training and internal validation. The test set constitutes an independent external validation set according to the validation strategy described in OECD Guidance [52].

As shown in Figure 1, after the splitting, the molecular descriptors in each training set were filtered to exclude uninformative and redundant features, potentially leading to numerical instability. This procedure reduces the complexity of the data used to develop and validate the models. For this purpose, an R-based algorithm recently published [53] was applied. The algorithm excludes descriptors with low variance (i.e., same value for more than 80% of the compounds), pairwise correlations greater than 0.95, and those with a range spanning more than two orders of magnitude.

### 2.3. QSAR Models Development

Multiple Linear Regression (MLR) using the Ordinary Least Squares (OLS) method was employed as the modelling algorithm. Variable subset selection was performed by identifying the best combinations of variables through the step-up procedure. This procedure, originally proposed by Rücker and colleagues [54], was implemented in our laboratory as an in-house R script [53]. The step-up procedure can be considered a variant of stepwise selection [54] and was preferred over other variable-selection methods (e.g., genetic algorithms) because its performance is similar but at a much lower computational cost. In this study, the step-up procedure generated populations of MLR models, ranked according to their coefficient of determination (R^2^). The search for the best populations of models was conducted by exploring variable subsets ranging from 1 to 10 descriptors.

Furthermore, to determine the level of complexity (i.e., number of descriptors selected in the models) that provides the best compromise between fitting ability and control of overfitting, the leave-one-out bootstrap procedure [55], was applied. This approach enabled the quantification of the bootstrapped mean absolute error (MAE_boostrap_). A plateau or an increase in MAE_boostrap_ with an increasing number of descriptors was interpreted as an indicator of potential overfitting. In addition, the agreement between MAE bootstrap and MAE calculated for the external validation sets provided additional proof of the expected predictive ability of the models. Furthermore, it demonstrated that the splitting strategy did not affect the predictive performance of the proposed QSARs. Once the most appropriate population of models was identified, the applicability domain of the training set was investigated by plotting hat values vs. standardized residuals, together with the analysis of potential outliers based on the standardized residuals calculated for each prediction [56]. In this work, a compound (in the training set) was considered a potential outlier, in a population of 25 models with a specific complexity, if its standardized residual exceeded the value of 2.5 in most of the models within the population. If the presence of outliers was linked to predictive instability, i.e., with overfitting appearing at low complexity, then chemicals consistently detected as outliers were excluded from the respective dataset and the entire modelling procedure was repeated. Additionally, a dedicated analysis was performed to evaluate both the molecular structures and the experimental values, in order to understand their anomalous behaviour.

Moreover, the randomization of the descriptors [54] within their range of values, both considering the nature of the descriptors (i.e., discrete, continuous, binary) or not [53] was performed to minimize the probability of coincidental relationships between the response and the descriptors. The randomization was performed by repeating the step-up procedure 100 times. The evaluation of the model fitting and internal robustness was conducted by using several metrics, i.e., R^2^, MAE, and leave-one-out cross-validated R^2^ (Q^2^_LOO_). Furthermore, the Y-scrambling procedure (50 iterations) was carried out to evaluate chance correlation between the descriptors and the response of the selected model, in terms of the average of R^2^ (R^2^_YS_). Low R^2^_YS_ values are observed when the likelihood of chance correlation, among the model descriptors and the response, is also low. Additional details regarding the formulae used to calculate the regression metrics listed above are provided in Supplementary Materials File S2.

The best model for each endpoint was chosen based on fitting and robustness metrics, i.e., choosing among the models that had the lowest average bootstrap MAE.

### 2.4. External Validation and Applicability Domains

After the selection of the best model for each endpoint, their predictive performance models were assessed using external test sets obtained through the splitting procedure described above. The external predictive performance was quantified based on predictions generated for the test set. The metrics used for the evaluation of external validation are the MAE and Q^2^_EXT_; the related formulae are provided in Supplementary Materials File S2.

### 2.5. Applicability Domain

According to the ECHA guidelines on QSAR methodologies, models must have a clearly defined applicability domain (AD) to ensure the reliability of their predictions. Predictions falling outside this domain are considered unreliable and should not be used to support regulatory decisions or risk assessments [44,57]. To date, there is no unique method for defining the applicability domain, as it can vary depending on the characteristics of the dataset and the model development methodology [58].

Linear regression models are among the most interpretable and user-friendly approaches, partly because they allow for a well-established and clearly defined applicability domain based on the chemical properties of the substances in the training dataset. In this study, for each model, the applicability domain was defined using information derived from the chemical structures of the training-set compounds and their experimental endpoint values. The structural AD of QSAR models was quantified using the leverage approach, supported graphically by the Williams plot, to identify both structural and response AD information. In the Williams plot, the x-axis reports the leverage values (i.e., the diagonal elements of the hat matrix; Equation (1)), whereas the y-axis reports the standardized residuals of each compound. The standardized residuals are calculated as described in Supplementary Materials File S2. In more detail, on the x-axis, the leverage values quantify the distance of each compound from the centroid of the model and reflect its influence on the regression. The threshold value h^*^ is defined as 3×(p+1)/n, where *p* is the number of descriptors included in the model and *n* is the number of training compounds. Chemicals with leverage values greater than h^*^ were considered structural outliers, as predictions for such compounds become increasingly unreliable with increasing leverage.

The hat matrix (or influence matrix) used to calculate leverage values is defined as

(1) $$\mathrm{HAT}=X(X^{T}X{)}^{-1}X^{T}$$

where *X* is the n×p data matrix.

On the y-axis, the standardized residuals measure how well the model predicts the response for each compound. Compounds with standardized residuals exceeding ±2.5 standard deviation units were classified as outside the response AD.

In addition to the leverage-based assessment, the reliability of each prediction was evaluated by comparing its prediction interval with the maximum and minimum uncertainty estimated for the model’s training set (as calculated in Supplementary Materials File S2). Predictions were considered reliable when their associated uncertainty fell within the uncertainty range defined for the training set and when their predicted values were within the experimental response range observed in the training set. Details on the calculation of prediction intervals are provided in Supplementary Materials File S2.

### 2.6. Case Study

Models generated in this study for RE and the GWP_100yr_ were applied to a large dataset including more than 13,000 chemicals and possible alternatives to refrigerants, obtained by integrating data reported in the literature [26,59] using the QSAR ME-Profiler 2025 [60]. As the models were developed using descriptors calculated by the software Mordred version 1.2.0, descriptors’ names were changed to match those included in QSAR-ME Profiler 2025. Name conversions are provided in Table S2. QSAR-ME Profiler 2025 allows for the direct examination of predictions through graphical visualization, applicability-domain assessment, prediction uncertainty analysis, and structural similarity evaluation.

Betowski and colleagues in their publication [26] listed more than 1200 substances for which RE values were estimated using computational chemistry techniques and the model of Pinnock [61]. SMILES notations for these chemicals were retrieved from EPA Dashboard [48] and PubChem databases [49] using the CAS number, chemical name, and molecular formula provided for each substance. SMILES notations were subsequently canonicalized as described in the previous section prior to calculating the molecular descriptors and applying the QSARs. Geng and colleagues [59] used the data from the latest WMO assessment report [22] to develop QSAR models for the GWP and ODP endpoints. The authors developed three distinct models: two models for the GWP endpoint, one regression-based and one classification-based. The classification model divides GWP into six categories: (0, 1), [1, 30), [30, 100), [100, 300), [300, 1000], and (1000, +∞). The values of GWP in the regression model are expressed as ln GWP. Unlike the present work, however, they adopted several linear and non-linear modelling techniques (i.e., RF, XGB, SVR, ANN, MLR, LASSO and RR) based on 3D molecular descriptors, among others, characterized by lower interpretability, and they did not include an analysis of the applicability domain.

These models were applied by Geng and colleagues [59] to screen a large dataset of potential sustainable alternative refrigerants which included more than 12,000 organic compounds selected on the basis of their structural similarity to the WMO substances. The SMILES notations available for these substances were canonicalized in order to calculate the molecular descriptors and apply the here proposed QSARs.

## 3. Results and Discussion

Following the methodology described above, 72 substances with no SMILES notation were eliminated before SMILES canonicalization, and an additional 38 duplicates, salts or mixtures were eliminated from the dataset after SMILES canonicalization. The data curation procedure led to the creation of a final dataset consisting of 672 compounds, divided into 21 classes, mostly halogenated, listed in Supplementary Materials File S1 (Sheet 01).

Among these substances, the most represented classes are hydrochlorofluorocarbons (264 out of 672, 39%) followed by hydrofluorocarbons (107 out of 672, 16%) and halogenated ethers (71 out of 672, 11%). Additional information related to the distribution of chemicals within the other classes is reported in Table S3 (Supplementary Materials File S2).

### 3.1. Global Warming Potential—QSAR

The GWP_100yr_ dataset after data curation consisted of 539 chemicals. Specifically, 132 chemicals were removed having GWP_100yr_ values < 1 or <<1. Details on chemicals discarded from the modelling, divided by class, are reported in Table S4 (Supplementary Materials File S2). Experimental GWP_100yr_ values in this dataset range from a minimum of 1 to a maximum of 32,300. The dataset used for QSAR development is reported in Supplementary Materials File S1. The dataset splitting resulted in 405 substances in the training set and 134 substances in the test set.

Molecular descriptors were calculated (total of 2493) and subsequently filtered to exclude redundant and not informative variables, resulting in a final set of 164 descriptors. A population of models including from 1 to 10 molecular descriptors was generated using the step-up method. A total of 25 models were retained for each level of model complexity for a total of 825 models. The analysis of the MAE_BOOTSTRAP_ behaviour in the population of models (Figure S1, Supplementary Materials File S2) highlighted a flattening of the MAE curve and an increase in the standard deviation calculated for the metric in bootstrapped models including six or more descriptors, suggesting instability due to outliers or overfitting above that level of complexity. Following a conservative approach, models including up to four variables, with a maximum R^2^ = 0.65 were selected for further investigation. A total of ten chemicals consistently detected as outliers among models with four variables were identified and removed according to the procedure described in the Methods section. Specifically, ID 379 (1-Fluorohexane), ID 385 ((Z)-HFO-1336mzz), ID 386 (HFO-1447fz), ID 388 ((E)-HFO-1438ezy), ID 438 ((E)-Perfluoro-2-butene), ID 457 (HFE-263mf), ID 479 (1,1,2,2-tetrafluoro-3-methoxy-propane), ID 517 (Perfluorobutyl acetate), ID 518 (Perfluoropropyl acetate) and ID 475 (HFE-356mmz1) were excluded from further model development. GWP values for these chemicals are commented on in Supplementary Materials File S2 (Table S5). Further investigation of these compounds (Supplementary Materials File S2, Table S5) showed that they either exhibited structural features not represented by the remaining compounds within the same chemical class or had experimental GWP values inconsistent with those reported for structurally similar compounds. These chemicals were associated with large prediction residuals (i.e., they were detected as outliers) in 23 to 25 of the 25 independently generated models at the selected level of optimal complexity within the population of models generated by the step-up procedure. This result supports their classification as systematic outliers rather than model-specific outliers. Detailed information for each compound is provided in Supplementary Materials File S2. After cleaning the dataset from these outliers, a new splitting, filtering and modelling procedure was carried out. The final dataset consisted of 397 compounds in the training set and 132 in the test set. The new model populations were analyzed, and the number of variables was selected after evaluation of the behaviour of the MAE_BOOTSTRAP_ metric within the new population of models. In particular, Figure S2 shows a clear improvement of the model accuracy after removal of the outliers compared to SE and MAE_BOOTSTRAP_ values reported in Figure S1, and the absence of overfitting in the studied population, up to 10 variables.

**Figure 2:**
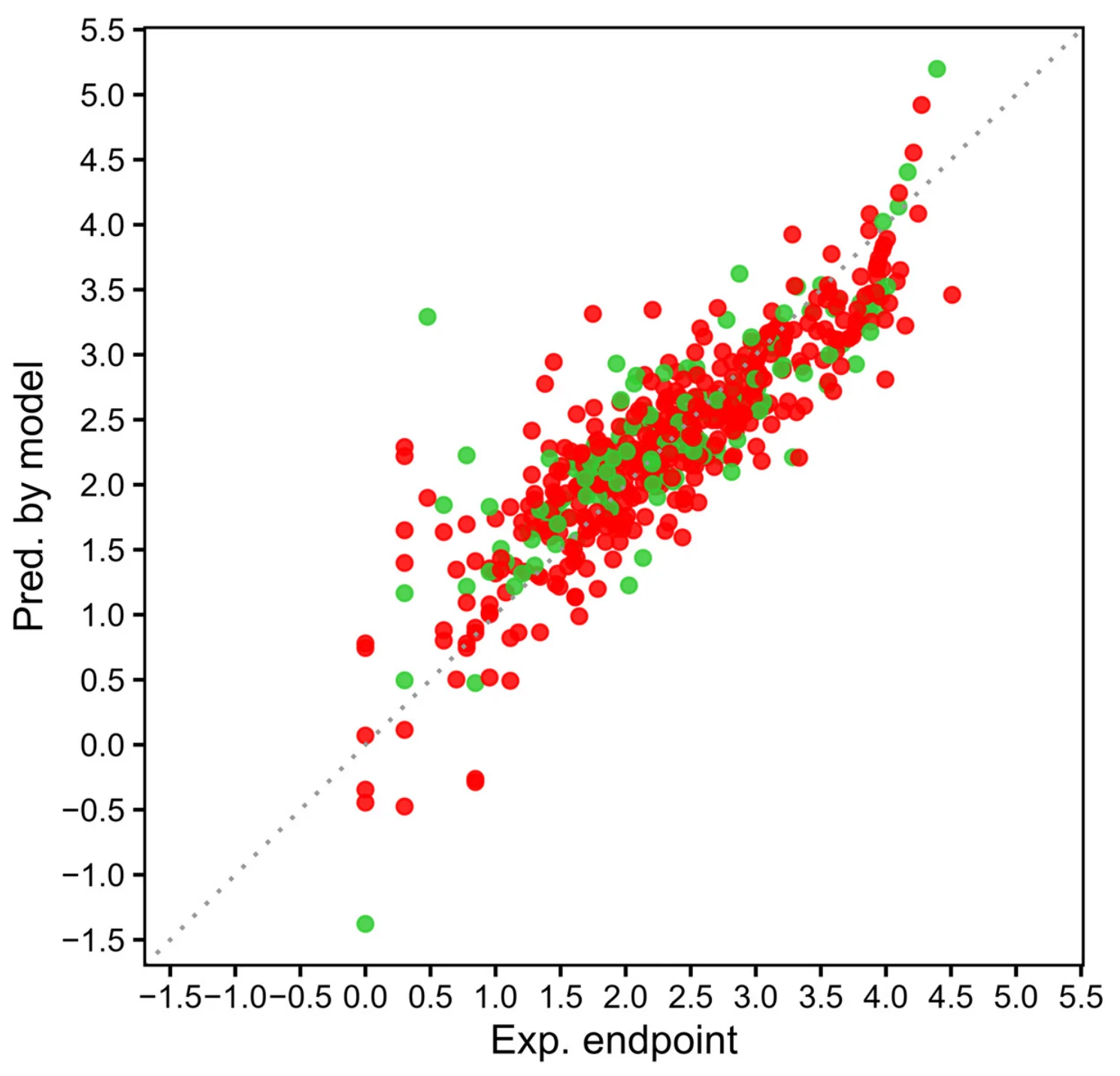
Regression diagnostic plots for the MLR-QSAR model: comparison between experimental (Exp. endpoint) and model predicted (Pred. by model) values for GWP endpoint. Red points represent the training-set substances, while green points represent the test-set substances.

To preserve the model’s quality as well as interpretability, the best-performing model was selected from those based on four variables. This choice was done taking into account the standard error within the models at the same level of complexity, as well as the small decrease in MAE_BOOTSTRAP_ in models with a complexity above four, different metrics (see Table 1), and the inspection of regression diagnostic plots (Figure 2 and Supplementary Materials File S2).

The equation of the best model based on four molecular descriptors (i.e., ETA_epsilon_5, SpMAD_A, GATS2dv and AETA_eta_L) trained on the splitted dataset (Equation (2)) is reported below and the plot of experimental results versus predictions are reported in Figure 2, while the values of the statistical metrics are summarized in Table 1.

(2) $$\begin{array}{c} {\mathrm{logGWP}}_{100\mathrm{yr}}=-3.43\left(\pm 1.28\right)+6.38\;\left(\pm 0.56\right)\times \mathrm{ETA}\;\_\;\mathrm{epsilon}\;\_\;5-3.97\;\left(\pm 0.68\right)\times \mathrm{SpMAD}\;\_\;A\;\\ +0.75\;\left(\pm 0.16\right)\times \mathrm{GATS}2\mathrm{dv}+2.67\;\left(\pm 1.10\right)\times \mathrm{AETA}\;\_\;\mathrm{eta}\;\_\;L \end{array}$$

An investigation of the regression diagnostic plots (reported in Supplementary Materials File S2) highlights several relevant aspects of the model performance. The experimental versus predicted response values (Figure 2) show that most data points are regularly distributed along the diagonal, indicating good overall agreement between predicted and experimental logGWP_100-yr_ values. However, compounds characterized by low logGWP_100-yr_ values exhibit a less regular distribution and are more difficult to predict accurately. This behaviour has already been reported in the literature, where low GWP substances are identified as intrinsically more challenging to predict and associated with higher uncertainty compared to higher GWP compounds [17,62]. Our model shows a tendency toward overestimation rather than underestimation of GWP values.

The model includes 16 distinct chemical classes, as illustrated in Figure S3. Hydrochlorofluorocarbons, which represent the most abundant class in the dataset, are mainly distributed around intermediate GWP values. In general, all the classes with more than three points appear reasonably well aligned along the diagonal, whereas halogenated ethers and ketones (intermediate GWP values) and chlorofluorocarbons (high GWP values) present some deviations. This last class occupies the upper region of the plot (Figure 2), together with fully fluorinated species, reflecting their higher GWP values compared to the rest of the dataset. At the lower end of the GWP scale, bromocarbons, hydrobromocarbons, and halons, as well as unsaturated chlorocarbons and hydrochlorocarbons, dominate the graph and do not present extreme deviation from the diagonal.

The residuals plot (Figure S4) shows a general random distribution of the residuals above and below the zero mostly spanning between ± 1 with some exceptions at the extremes of the range. Furthermore, some outliers are identified in the central part of the graph, as expected considering what was mentioned above for halogenated ethers and ketones.

The analysis of the standardized residuals vs. leverages (Figure S5) indicates that 22 compounds, including 3 from the test set and 19 from the training set, fall outside the model applicability domain, defined by leverage approach of *h* > h* = 0.038. Among these compounds, three substances were simultaneously identified as potential response outliers. Two of them, belonging to the training set, minimally exceed the standardized residual cut-off threshold fixed at ±2.5 (ID_GWP 390 -Carbon tetrachloride- and 404-p-chlorobenzotrifluoride-). In addition, 12 compounds were identified as potential outliers. For all the substances in the dataset, the descriptor values, standardized residuals, leverage values, and both experimental and predicted response values are summarized in Supplementary Materials File S1, Sheet 03.

Among the 22 compounds located outside the structural applicability domain, the following class distribution was observed: six bromocarbons, hydrobromocarbons and halons; four fully fluorinated species; two chlorocarbons and hydrochlorocarbons; two unsaturated chlorocarbons and hydrochlorocarbons; two with no details about class; two special compounds; one unsaturated chlorofluorocarbon and hydrochlorofluorocarbon; one oxygenated hydrocarbon; one hydrochlorofluorocarbon; and one hydrofluorocarbon.

Despite their high leverage, these compounds were retained in the dataset in order to keep the applicability domain as broad as possible.

Fifteen compounds were identified as outliers in the Williams plot (Figure S5 and Table S6). To further investigate their behaviour, these substances were analyzed by PCA generated using the descriptors selected in model 1 as the input variables (i.e., ETA_epsilon_5, SpMAD_A, GATS2dv and AETA_eta_L). As shown in Figure S6 in Supplementary Material S2, among the response outliers (highlighted in circle red and listed in Table S6), only two compounds, ID_GWP 390 (Carbon tetrachloride) and 400 (1,4-dichlorobenzene), are clearly separated from the rest of the dataset. These two compounds are in fact also located outside the structural applicability domain of the model (Figure S5). The remaining response outliers are distributed within the structural space of the model, typically positioned among compounds characterized by medium or high GWP values despite having low experimental values. Consequently, they are predicted similarly to their nearest neighbours, suggesting that the selected descriptors are not fully sensitive to subtle structural features responsible for reduced GWP. These compounds belong, for example, to the classes of unsaturated chlorocarbons and hydrochlorocarbons, unsaturated chlorofluorocarbons and hydrochlorofluorocarbons, halogenated ketones and halogenated alcohols. These classes are poorly represented in the dataset, which limits the model’s ability to learn the relevant structural patterns required for accurate prediction. A comparable limitation was also reported in the study of Devotta and colleagues [17]. A detailed compound-by-compound analysis, including PCA positioning and standardized residual values, is provided in the Supplementary Materials File S2 (Figures S6–S9 and Table S6).

Removing the response outliers belonging to the training set does not lead to an improvement in model predictivity. As expected, model fitting improves, with an internal training R^2^ = 0.81, however, while the external fitting (R^2^ = 0.69) remains essentially unchanged, the external predictivity (Q^2^_EXT_) slightly decreases from 0.68 to 0.66 while the MAE_EXT_ increases from 0.36 to 0.38. Therefore, excluding these compounds from model construction cannot be justified, as it results in the loss of potentially informative chemical space without any meaningful improvement in predictive performance.

The most influential descriptor according to its standardized coefficient (Supplementary Materials File S2, Table S7) is ETA_epsilon_5, which shows a positive sign in the equation of the model (Equation (1)). Interestingly this descriptor has also been identified as highly relevant in a previous study by Geng and colleagues [59]. This descriptor is an Extended Topochemical Atom [51] and reflects the electronic environment and the chemical activity of the molecule. In the present study, higher values of ETA_epsilon_5 are associated with higher GWP values, as clearly shown in the PCA analysis (Figures S7–S9). The second most influential descriptor is SpMAD_A, which is calculated from the adjacency matrix scaled by the number of atoms and has a negative coefficient in regression equation 1. SpMAD_A is a measure of the average molecular connectivity, calculated from the adjacency matrix and normalized on the molecular size [63,64,65]. GATS2dv is the third most influential descriptor, which is a Geary autocorrelation descriptor at topological lag 2 weighted by valence electrons. This descriptor captures local heterogeneity in the distribution of valence electrons along the molecular graph and reflects the similarity between atom pairs separated by two bonds [51,66]. The fourth descriptor in terms of importance is AETA_eta_L, belonging to the Etacomposite Index family [51]. This descriptor accounts for the global electronic extension and topological connectivity of the molecule, excluding explicit hydrogen contributions. In the present dataset, higher values of AETA_eta_L are associated with lower GWP values. Compounds characterized by high AETA_eta_L typically exhibit less branched structures and have a lower degree of halogenation and hydrogen bonds, features commonly linked to lower persistence and climate impact [32]. PCA results shown in Figures S7–S9 further support these observations.

Notably, these descriptors have already been identified as relevant for describing GWP-related phenomena in previous studies. For example, in the models developed by Geng et al., SpMAD_A, AETA_eta_L, and ETA_epsilon_5 were selected in both regression and classification frameworks, among 44 descriptors used for regression and 73 for classification [59].

### 3.2. Radiative Efficiency–QSARs

Within the original dataset, a total of 670 compounds were identified as characterized by experimental removal efficiency data suitable for the development of QSARs. The dataset was divided into a training set of 503 compounds and a test set of 167 compounds. The modelled dataset is highly heterogeneous, encompassing 20 distinct chemical classes.

The experimental values for the modelled endpoint (Log RE) span from −2.52 to 0.26, with a mean value of −0.74.

The modelling results show that the populations of models obtained across increasing model complexity are highly stable. As evidenced by the average bootstrapped MAE trend (Figure S13 in Supplementary Materials File S2), no pronounced oscillations or abrupt changes are observed when increasing the number of variables from 1 to 10. This behaviour indicates that the predictive performance of the models remains robust across different levels of complexity and that no clear signs of overfitting emerge within the explored variable range. Overall, the bootstrap analysis supports the stability and reliability of the developed model populations.

**Figure 3:**
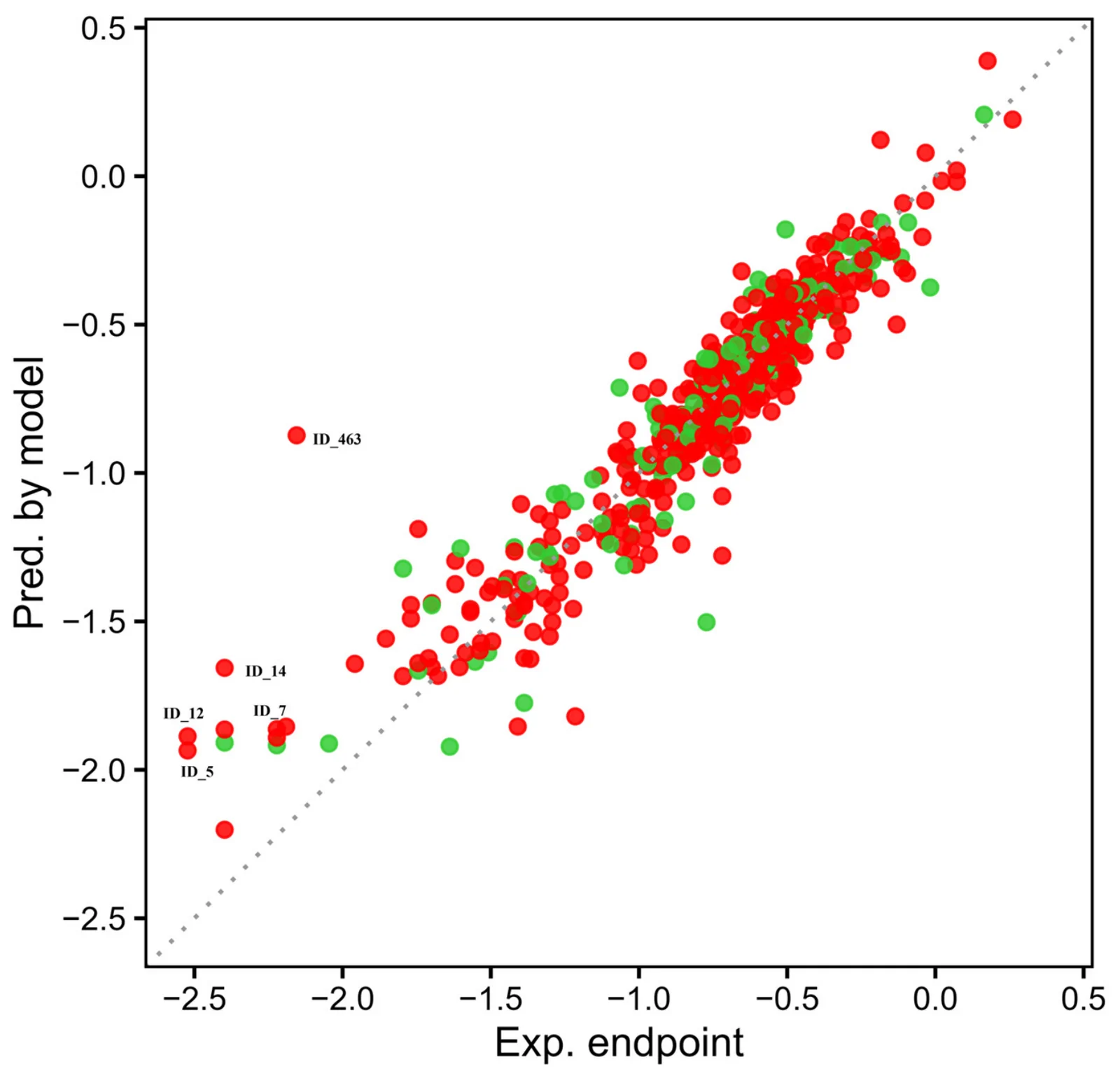
Regression diagnostic plots for the MLR-QSAR model: comparison between experimental (Exp. endpoint) and model-predicted (Pred. by model) values for RE endpoint. Red points represent the training-set substances, while green points represent the test-set substances.

The optimal model complexity was identified at four variables, as the average MAE_BOOSTRAP_ variation is very limited after this level of complexity. The analysis of models based on four variables led to the selection of the best-performing QSAR, which is based on the molecular descriptors CIC1, SpMAD_Dzp, SsCH3, and JGT10. The regression equation of the selected model based on the splitted dataset (Equation (3)) is reported below as well as the statistical performance metrics (Table 2). The experimental versus predicted plot, is provided in Figure 3.

(3) $$logRE=-1.66\left(\pm 0.03\right)+1.04\;\left(\pm 0.09\right)\times JGT10+0.06\;\left(\pm 0.0067\right)\times SpMAD\;\_\;Dzp-0.13\;\left(\pm 0.03\right)\times CIC1-0.08\;\left(\pm 0.02\right)\times SsCH3$$

Inspection of the experimental versus predicted plots (Figure 3) reveals that, in the lower tail of the chart, some outliers can be identified and their related Log REs tend to be overestimated.

The class-wise regression plots reported in Supplementary Materials File S3 further highlight this behaviour. This figure shows that some classes among the several overlapped in the lower region of the regression space, and in particular hydrocarbons, oxygenated hydrocarbons, and special compounds, present larger deviations from the expected behaviour. This could be interpreted as related to the simplicity of the model, which is based on only four molecular descriptors, making the model relatively less sensitive to capture the structural information related to RE values of less represented chemicals classes or to chemicals which are structurally different from the rest of the data set (the left and lower region of the model is occupied mostly by chemicals that are not halogenated hydrocarbons). However, on the other hand, it is important to highlight that the model has a good fitting and is in general robust and predictive; the aforementioned overestimations of the RE are for chemicals which are not expected to have a large impact on climate (i.e., low persistency, low RE and low GWP).

Analysis of the overestimated training compounds, ID_RE 463 (1,1-dichloro-2,2-difluoro-ethene), 14 (Formaldehyde), 12 (Benzene), 5 (Propane, R-290), and 7 (Isobutane, R-600a), shows that all of them are identified as response outliers in the Williams plot (Figure S15) and are located outside the structural applicability domain, with the exception of ID_463 (1,1-dichloro-2,2-difluoro-ethene), which is classified exclusively as a response outlier. Furthermore, values reported in Supplementary Materials File S1 (Sheet 05) indicate that 34 compounds fall outside the structural applicability domain. Among these, only 10 compounds are simultaneously identified as response outliers.

These high leverage compounds belong to a wide range of chemical classes, specifically eleven hydrocarbons, seven fully fluorinated species, four hydrofluorocarbons, three halogenated ethers, two oxygenated hydrocarbons, two iodocarbons, two special compounds, and two chlorocarbons and hydrochlorocarbons, as well as individual representatives of bromocarbons, hydrobromocarbons, and halons. This distribution reflects the structural heterogeneity of the dataset.

Furthermore, 16 compounds were identified as response outliers (listed in Supplemental Material S2, Table S8), 10 of which are also classified as high leverage compounds. PCA analysis confirms that most of these molecules are associated with low experimental Radiative Efficiency values. In particular, the problematic region is in the low response range, with experimental values spanning from −2.52 to −0.72.

According to the standardized coefficient of Equation (2) (Supplementary Materials File S2, Table S9), the most influential descriptor is JGT10, which contributes positively to the regression equation. JGT10 is a Geary autocorrelation descriptor at lag 10 belonging to the GETAWAY (GEometry, Topology, and Atom-Weights AssemblY) family. It describes the spatial distribution of atomic properties across topological distance within the molecule [51,63]. The PCA analysis (Figures S16–S18 in Supplementary Material S2) shows that PC1, explaining approximately 52% of the total variance, represents the main gradient associated with Radiative Efficiency. PC1 is primarily influenced by JGT10 and SpMAD_Dzp, the latter being the second most influential descriptor in Equation (2) and contributing positively to the model. SpMAD_Dzp describes the spectral mean absolute deviation from Barysz matrix weighted by polarizabilities. The Barysz matrix is a weighted distance matrix that accounts for multiple bonds and heteroatoms [51,67]. The combination of higher values of SpMAD_Dzp and JGT10 reflects high values of RE. The third descriptor in terms of influence is CIC1, which shows a negative contribution to the equation. CIC1 belongs to the complementary information content family and describes the information of primary importance regarding the connectivity of the molecular graph [51]. Finally, SsCH3, also with negative contribution, belongs to the estate descriptor family and represents the total number of methyl groups connected with a single bond [51,68].

In order to use all the experimental and structural information available in both the training and test sets modelled so far, the MLR-QSAR models developed for ${\mathrm{logGWP}}_{100-\mathrm{yr}}$ and LogRE were recalibrated on the corresponding full datasets. The equations of the full models are reported in Supplementary Material S2, together with the statistical metrics used to evaluate model fitting, robustness, and predictive reliability.

As expected, the full models exhibit regression coefficients consistent with those calculated for the models developed using the splitted datasets, as well as presenting comparable statistical performance and applicability-domain characteristics. This confirms the stability of the descriptor selection and the robustness of the modelling strategy across different calibration schemes.

The prediction and the uncertainty values for the full models are reported in the Supplementary Materials File S1, while the corresponding diagnostic plots (including Residual and Williams plots) are provided in Supplementary Materials File S2.

#### Case Study: Application of the New QSARs to Screen Sustainable Alternatives

In order to show the utility of the new models for screening purposes, a total of 13,509 SMILES notations compiled from previous literature works [26,59], which address the evaluation of chemicals and possible alternatives to refrigerants, were collected and canonicalized. The aim of this case study is to identify and prioritize compounds with potential safe behaviour (i.e., low GWP) based on the molecular structure using a consensus approach which involves independent models, i.e., the new models developed in this study and those developed in the previous literature works [26,59].

The molecular descriptors required for the application of the developed models were calculated as described in the methods section. Three compounds (ID_12760 Heptafluorotetrahydro(nonafluorobutyl)furan, ID_13241 Methane, ID_13246 Methane isocyano -) were excluded from the GWP screening due to invalid results in the calculation of the molecular descriptors. Regarding the application of the RE model, ID_12760 and ID_13246 were also excluded for the same reason described above, while ID_13241 fell outside the model AD.

The full model equations recalibrated for Log GWP and Log RE reported in the previous section were applied to 13,506 and 13,507 chemicals respectively, through the software QSARME-Profiler 2025 [60]. The software allowed for the exploration of the AD of the models, thereby identifying compounds falling outside the structural applicability domain and/or outside the experimental range of the two responses. Results of the screening considering the evaluation of the AD and the reliability of the predictions are summarized in Table 3. Only predictions generated within the applicability domain of the new models were included in the case study to ensure reliability.

For this reason, the inclusion in the AD of predictions generated using the new models provides important information on the reliability of these predictions compared to those obtained using the model proposed Geng and colleagues [59], rather than serving as an additional information for a weight-of evidence approach against a reference model. In fact, these authors did not explicitly evaluate the applicability domain for their models. Furthermore, the literature model is a non-linear QSAR built using 44 descriptors, whereas the new model presented here is linear and based on only four variables.

Furthermore, the high correlation between RE values predicted for 1098 chemicals by the new model (inside the AD) and empirically calculated RE [26] demonstrates the strong consistency between the two approaches and corroborates the QSAR predictions.

**Figure 4:**
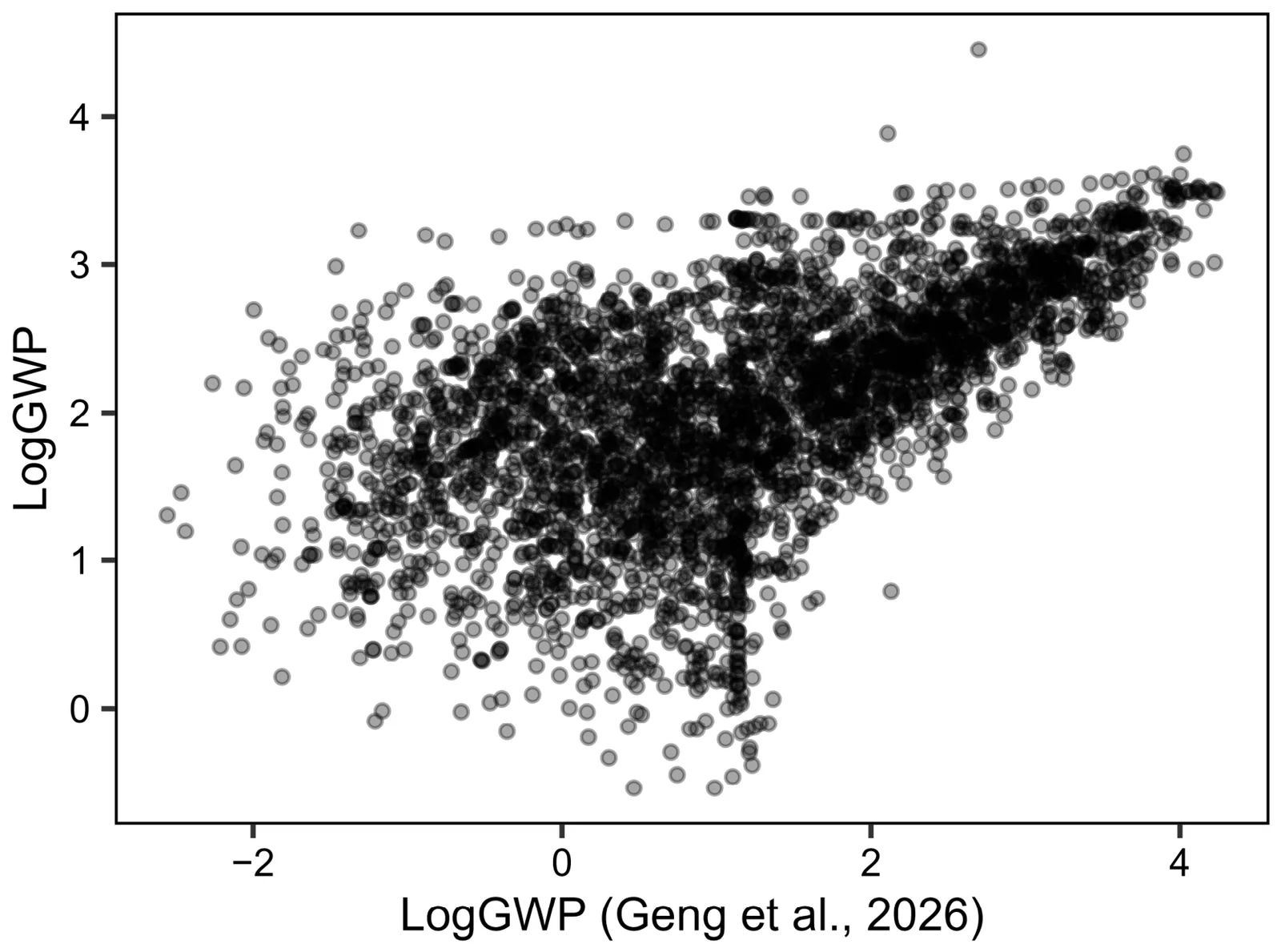
Comparison of LogGWP values predicted by the model developed in this study (within AD) (y-axis) against predictions obtained from the non-linear model of Geng et al. [59] (x-axis) for the compounds included in the database (n = 3569); darker points correspond to higher density of overlapping chemicals.

The correlation (r = 0.56) between predictions by Geng’s model and those generated in the applicability domain of the new QSAR for log GWP shows a fair agreement among the two models, especially for chemicals with high GWP values. However, Figure 4 shows that the region of greatest disagreement corresponds to compounds with low GWP values. This behaviour is consistent with the tendency of the new QSAR model to overestimate predictions in the low-GWP range, as discussed above. Since no experimental values are available for these compounds, these differences cannot be used to establish the superiority of either model.

**Figure 5:**
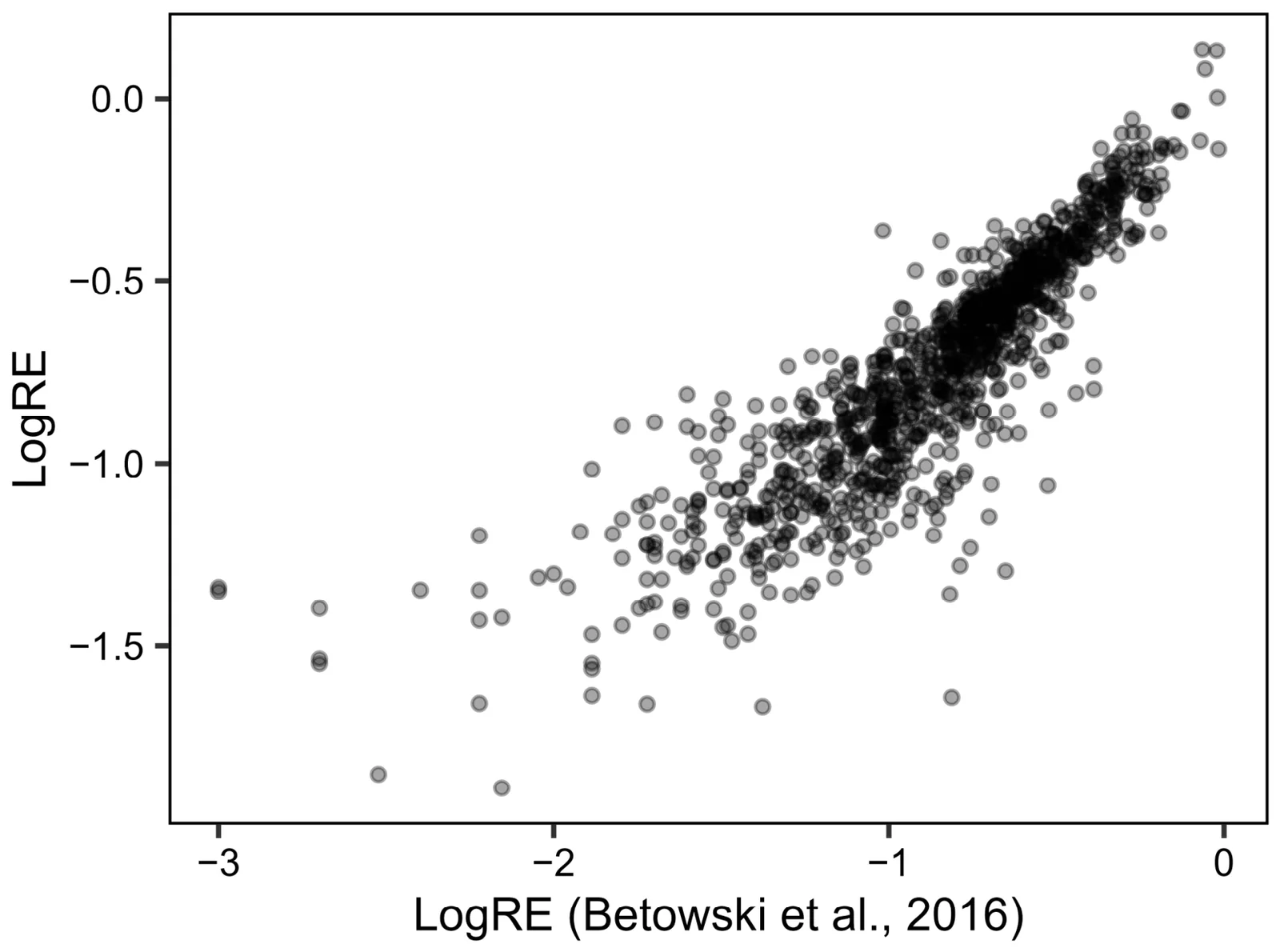
Comparison of LogRE values predicted by the model developed in this study (within AD) (y-axis) against predictions (x-axis) generated by Betowski et al. [26] for the compounds included in the studied dataset (n = 1098). Darker points correspond to higher density of overlapping chemicals.

Applying the same approach to the available Radiative Efficiency (RE) data, a similar pattern emerges but the correlation is stronger (r = 0.92): the greatest discordance between predicted and reference values is observed in the low-RE region (Figure 5). However, it must be noted that the majority of these chemicals belong to the bromocarbons, hydrobromocarbons, and halons chemical classes, and/or chemicals containing nitrogen atoms, and the model developed by Betowski [26] is known to systematically overestimate the Radiative Efficiency for these categories.

To show how two independent in silico approaches can be directly relevant to the screening of safer chemicals, we performed a comparative analysis using the screening threshold of GWP < 100 rather than continuous prediction values. Among the 3569 chemicals falling within the applicability domain of the new log GWP model, 1552 chemicals (43%) were classified as low GWP by both the literature and the new model, identifying a potential set of candidate sustainable alternatives supported by independent predictions. Conversely, 927 chemicals (27%) were consistently classified as high GWP. For the remaining 1090 chemicals there is not an agreement, and disagreements are not balanced. In more detail, fifty compounds were classified as GWP < 100 by the new model and ≥100 by the literature model [59]; 1040 chemicals were classified as ≥100 by the new model and <100 by the literature model [59]. These results demonstrate that the here proposed approach is more conservative than the one proposed by Geng and colleagues [59]. This is consistent with what was already discussed above.

The dataset reported in Supplementary Material S4 provides integrated information for more than 13,000 substances, enabling the identification of 1552 potential candidate sustainable alternatives characterized by reliable predictions falling within the structural AD of the proposed models, and GWP values consistently predicted below 100 by the two models. These chemicals represent priority candidates for further investigation. Although experimental validation would be required to verify the actual performance of the models, the consensus between independent approaches and the analysis of the applicability domain provides additional confidence in their reliability and a rational basis for the prioritization of chemicals in the absence of experimental data. Additionally, all the substances predicted within the applicability domain are considered reliable predictions and may support preliminary LCA and prioritization of candidate chemicals.

## 4. Conclusions

In this study, QSAR models were developed for the prediction of climate indicators from the molecular structure and applied as screening tools to support the in silico identification of alternatives to unsafe chemicals. The modelling strategy was designed to reflect established regulatory practices, ensuring compliance with the OECD principles for QSAR model validation [45], including transparent model definition, applicability-domain characterization, and explicit consideration of prediction reliability. It should be emphasized that the new models predict Global Warming Potential (100-years time horizon) and Radiative Efficiency as properties related to the molecular structure. Additional analysis involving chemical emission rate, production volume, use pattern and environmental release are outside the scope of the present work and were not performed. Accordingly, the proposed models are intended only to support the prediction of the climate indicators from the molecular structure, whereas the overall climatic impact of a substance requires the integration of these data with exposure information within a broader risk assessment framework.

The results demonstrated that the new models are useful to generate reliable predictions and to support climate-related impact assessment within the LCIA phase of LCA. In particular, QSAR predictions can support the preliminary assessment of substances for which experimental or atmospheric modelling data are unavailable, facilitating early-stage comparison of chemical alternatives and contributing to a more informed decision-making processes. Furthermore, the models were implemented in the QSAR ME-Profiler 2025 [60], enabling straightforward application and facilitating their practical use in sustainability assessment workflows. It is important to highlight that predictive modelling should be applied within a scientifically meaningful context. Prior to QSAR-based estimation, substances should undergo preliminary evaluation of their physicochemical properties to verify the relevance of predicting climate-related endpoints. Such pre-screening ensures that predictions are aligned with realistic use scenarios and prevents the inappropriate application of climate metrics to compounds outside the intended domain. A natural next step is to extend the same approach proposed in this work to other useful indicators such as the Ozone Depletion Potential and the Atmospheric Lifetime. Such, an expansion would enable a more comprehensive and exhaustive screening process, paving the way for the identification of sustainable alternatives. Additionally, the development of a dedicated model for low-GWP compounds may represent a relevant direction for future work. Given the intrinsic complexity associated with predicting very low GWP values and the limited availability of representative data, a dedicated investigation of such a model is beyond the scope of the present study.

In conclusion, the availability of the new QSAR models to predict GWP and RE supports the integration of in silico approaches within regulatory frameworks for both early-stage screening and the design of safer and sustainable alternatives to hazardous chemicals based solely on the chemical structure.

## Figures and Tables

**Table 1:** Summary of the statistical results of the GWP-QSAR. “Random Descriptors Range” and “Random Descriptors Typee” indicate the probability of coincidental relationships between the molecular descriptors and the response, using randomized descriptors within their numerical ranges (X-Random Descriptors Range), or considering both their numerical ranges and their nature, i.e., discrete, continuous, or binary (X-Random Descriptors Type). * 2.2 × 10^−14^ is the smallest representable number by the software.

	n	R^2^	MAE	Q^2^_loo_	Q^2^_EXT_	R^2^_YS_	MAE_BOOSTRAP_	X-Random Descriptors Range	X-Random Descriptors Type
Training	397	0.75	0.34	0.74		0.01	0.37 ± 0.001	<2.2 × 10^−14^ *	<2.2 × 10^−14^ *
Test	131	0.69	0.36		0.68				

**Table 2:** Summary of the statistical results of the RE-QSAR. “Random Descriptors Range” and “Random Descriptors Type” indicate the probability of coincidental relationships between the molecular descriptors and the response, using randomized descriptors within their numerical ranges (X-Random Descriptors Range), or considering both their numerical ranges and their nature, i.e., discrete, continuous, binary (X-Random Descriptors Type). * 2.2 × 10^−14^ is the smallest representable number by the software.

	n	R^2^	MAE	Q^2^_loo_	Q^2^_EXT_	R^2^_YS_	MAE_BOOSTRAP_	X-Random Descriptors Range	X-Random Descriptors Type
Training	503	0.87	0.10	0.86		0.01	0.10 ± 1.27 × 10^−4^	<2.2 × 10^−14^ *	<2.2 × 10^−14^ *
Test	167	0.88	0.09		0.89				

**Table 3:** Summary of the applicability-domain analysis for the external dataset. The table reports the number of compounds classified as outside the structural applicability domain, outside the prediction domain, and outside both domains for the GWP and RE models.

	GWP	RE
Out of the structural AD	1408/13,506	3930/13,507
Out of the range of the response	0/13,506	0/13,507
Out of the structural AD and the range of the response	7716/13,506	20/13,507

## Data Availability

All the data presented in this study are included in the article/Supplementary Materials, further inquiries can be directed to the corresponding author.

## Supplementary Materials

The following supporting information can be downloaded at https://www.mdpi.com/article/10.3390/toxics14080667/s1: Supplementary Materials File S1 (Sheet 00: the raw dataset containing only the endpoints of interest for this work; Sheet 01: final dataset after data curation; Sheet 02: modelled GWP dataset; Sheet 03: descriptor values, standardized residual, leverage values, and both experimental and predicted response values for all the substances in the GWP dataset (for both full and splitted model); Sheet 04: modelled RE dataset; Sheet 05: descriptor values, standardized residual, leverage values, and both experimental and predicted response values for all the substances in the RE dataset (for both full and splitted model).) Supplementary Materials File S2 (Table S1: Selected representative studies describing predictive approaches for estimating climate-related endpoints relevant to LCIA applications and chemical screening applications; definitions of the statistical metrics for the MLR-QSAR evaluation; definition of the standardized residuals; uncertainty quantification [17,26,27,32,35,39,41,59,61,69,70,71,72,73]; Table S2: Conversion table showing the differences in nomenclature between the Mordred descriptors and those in QSAR-ME Profiler 2025.; Table S3: Frequency in terms of number and percentage of the class of substances present in the dataset after curation, reported in the Supplementary Materials File S1 (Sheet 01); Table S4: Table reporting the quantities and the reasons why certain substances were excluded from the initial dataset of GWP endpoint, organized by substance class, to define the final GWP dataset, reported in Supplementary Materials File S1 (Sheet 02); Figure S1: Bootstrap analysis of the GWP-MLR models; Table S5: Substances considered as outliers for the dataset GWP; Figure S2: Bootstrap analysis of the GWP-MLR models (after the elimination of outliers); Figure S3: Regression diagnostic plots for the MLR-QSAR model (GWP endpoint) by class of chemicals; Figure S4: Plot of the residuals. Red points represent the training-set substances, while green points represent the test-set substances; Figure S5: Williams plot of the split MLR-QSAR model of the GWP endpoint; Table S6: Corresponding ID and Name of outliers and/or chemicals outside the structural domain of MLR-GWP; Figure S6: Plot of PC1 vs. PC2 from the PCA of the GWP model dataset; Figures S7–S9 show scores and loading of PC1 and PC1 from the PCA of the GWP model dataset; equation of GWP full model; Figures S10–S12: Diagnostic plots of GWP full model; Table S7: Standardized coefficients of the GWP model; Figure S13: Bootstrap analysis of the RE-QSARs; Figure S14: Plot of the residuals (RE model); Figure S15: Williams plot of the split MLR-QSAR model of the RE endpoint. Table S8: Corresponding ID and Name of outliers and/or chemicals outside the structural domain of MLR-QSAR model of the RE endpoint; Figures S16–S18 plots show scores and loading of PC1 and PC1 from the PCA of the RE model dataset; equation of RE full model; Figures S19–S21: Diagnostic plots of RE full model; Table S9: Standardized coefficients of the RE model.) Supplementary Materials File S3 (Regression diagnostic plots for the MLR-QSAR model (RE endpoint) by class of chemicals.) Supplementary Materials File S4 (Sheet 00: list of over 13,000 chemicals with their GWP and RE predicted values).

## Abbreviations

The following abbreviations are used in this manuscript:

SDGs	Sustainable Development Goals
QSAR	Quantitative Structure–Activity Relationship
EC	European Commission
CSS	Chemicals Strategy for Sustainability
SSbD	Safe and Sustainable by Design
LCA	Life Cycle Assessment
LCI	Life Cycle Inventory
LCIA	Life Cycle Impact Assessment
GWP	Global Warming Potential
ODP	Ozone Depletion Potential
IPCC	Intergovernmental Panel on Climate Chance
RE	Radiative Efficiency
WMO	Word Meteorological Organization
ANN	Artificial Neural Networks
SMILES	Simplified Molecular Input Line Entry System
MAE	Mean Absolute Error
MLR	Multiple Linear Regression
OLS	Ordinary Least Squares
AD	Applicability Domain
OECD	Organization for Economic Co-operation and Development

## References

[1] UN (2015). Transforming Our World: The 2030 Agenda for Sustainable Development.

[2] UN 17 Sustainable Development Goals (SDGs). https://sdgs.un.org/goals.

[3] European Commission (2019). The European Green Deal.

[4] European Commission (2020). Chemicals Strategy for Sustainability Towards a Toxic-Free Environment.

[5] Caldeira C., Farcal L.R., Garmendia Aguirre I., Mancini L., Tosches D., Amelio A., Rasmussen K., Rauscher H., Riego Sintes J., Sala S. (2022). Safe and Sustainable by Design Chemicals and Materials—Framework for the Definition of Criteria and Evaluation Procedure for Chemicals and Materials.

[6] Patinha Caldeira C., Farcal R., Moretti C., Mancini L., Rauscher H., Rasmussen K., Riego Sintes J., Sala S. (2022). Safe and Sustainable by Design Chemicals and Materials Review of Safety and Sustainability Dimensions, Aspects, Methods, Indicators, and Tools, EUR 30991 EN.

[7] Abbate E., Garmendia Aguirre I., Bracalente G., Mancini L., Tosches D., Rasmussen K., Bennett M.J., Rauscher H., Sala S. (2024). Safe and Sustainable by Design Chemicals and Materials-Methodological Guidance.

[8] Caldeira C., Garmendia Aguirre I., Tosches D., Mancini L., Abbate E., Farcal L., Lipsa D., Rasmussen K., Rauscher H., Riego Sintes J. (2023). Safe and Sustainable by Design Chemicals and Materials: Application of the SSbD Framework to Case Studies.

[9] (2006). Environmental Management—Life Cycle Assessment—Principles and Framework.

[10] (2021). Environmental Management—Life Cycle Assessment—Life Cycle Impact Assessment.

[11] Anastasab P., Lankey R. (2000). Life Cycle Assessment and Green Chemistry: The Yin and Yang of Industrial Ecology. Green Chem..

[12] Tabone M.D., Cregg J.J., Beckman E.J., Landis A.E. (2010). Sustainability Metrics: Life Cycle Assessment and Green Design in Polymers. Environ. Sci. Technol..

[13] Čuček L., Klemeš J.J., Kravanja Z. (2012). A Review of Footprint Analysis Tools for Monitoring Impacts on Sustainability. J. Clean. Prod..

[14] JRC (2010). International Reference Life Cycle Data System (ILCD) Handbook: Framework and Requirements for Life Cycle Impact Assessment Models and Indicators.

[15] JRC (2010). International Reference Life Cycle Data System (ILCD) Handbook: General Guide for Life Cycle Assessment: Detailed Guidance.

[16] Houghton J.T., Ding Y., Griggs D.J., Noguer M., van der Linden P.J., Dai X., Maskell K., Johnson C.A., IPCC (2001). Climate Change 2001: The Scientific Basis. Contribution of Working Group I to the Third Assessment Report of the Intergovernmental Panel on Climate Change.

[17] Devotta S., Chelani A., Vonsild A. (2021). Prediction of Global Warming Potentials of Refrigerants and Related Compounds from Their Molecular Structure—An Artificial Neural Network with Group Contribution Method. Int. J. Refrig..

[18] Masson-Delmotte V., Zhai P., Pirani A., Connors S.L., Péan C., Berger S., Caud N., Chen Y., Goldfarb L., Gomis M.I., Huang M., Leitzell K., Lonnoy E., Matthews J.B.R., Maycock T.K., Waterfield T., Yelekçi O., Yu R., Zhou B., IPCC (2021). Climate Change 2021: The Physical Science Basis. Contribution of Working Group I to the Sixth Assessment Report of the Intergovernmental Panel on Climate Change.

[19] Calvin K., Dasgupta D., Krinner G., Mukherji A., Thorne P.W., Trisos C., Romero J., Aldunce P., Barrett K., Blanco G., Lee H., Romero J. (2023). Contribution of Working Groups I, II and III to the Sixth Assessment Report of the Intergovernmental Panel on Climate Change. IPCC, 2023: Climate Change 2023: Synthesis Report.

[20] Solomon S., Qin D., Manning M., Chen Z., Marquis M., Averyt K.B., Tignor M., Miller H.L., IPCC (2007). IClimate Change 2007: The Physical Science Basis. Contribution of Working Group I to the Fourth Assessment Report of the Intergovernmental Panel on Climate Change.

[21] Young C.J., Hurley M.D., Wallington T.J., Mabury S.A. (2008). Molecular Structure and Radiative Efficiency of Fluorinated Ethers: A Structure-activity Relationship. J. Geophys. Res. Atmos..

[22] World Meteorological Organization (WMO) (2022). Scientific Assessment of Ozone Depletion: 2022, GAW Report No. 278, 509 pp..

[23] Bera P.P., Francisco J.S., Lee T.J. (2009). Identifying the Molecular Origin of Global Warming. J. Phys. Chem. A.

[24] Hischier R., Hellweg S., Capello C., Primas A. (2005). Establishing Life Cycle Inventories of Chemicals Based on Differing Data Availability (9 Pp). Int. J. Life Cycle Assess..

[25] Blowers P., Moline D.M., Tetrault K.F., Wheeler R.R., Tuchawena S.L. (2007). Prediction of Radiative Forcing Values for Hydrofluoroethers Using Density Functional Theory Methods. J. Geophys. Res. Atmos..

[26] Betowski D., Bevington C., Allison T.C. (2016). Estimation of Radiative Efficiency of Chemicals with Potentially Significant Global Warming Potential. Environ. Sci. Technol..

[27] Yang Z., Yang S., Wang X., Xiao J., Wang H. (2025). Prediction of Global Warming Potential for Gases Based on Group Contribution Method and Chemical Activity Descriptor. ACS Omega.

[28] Holmquist H., Lexén J., Rahmberg M., Sahlin U., Palm J.G., Rydberg T. (2018). The Potential to Use QSAR to Populate Ecotoxicity Characterisation Factors for Simplified LCIA and Chemical Prioritisation. Int. J. Life Cycle Assess..

[29] Douziech M., Oginah S.A., Golsteijn L., Hauschild M.Z., Jolliet O., Owsianiak M., Posthuma L., Fantke P. (2024). Characterizing Freshwater Ecotoxicity of More Than 9000 Chemicals by Combining Different Levels of Available Measured Test Data with In Silico Predictions. Environ. Toxicol. Chem..

[30] Schwirn K., Völker D., Løfstedt M., Fantke P., Bossa C., Sharma A., Posthuma L., Karakoltzidis A., Nikiforou F., Mikołajczyk A. (2025). The European Commission’s Safe and Sustainable by Design Framework: Bridging Innovation and Legislation. Environ. Sci. Eur..

[31] Gnyll L.R., Back D.D., Vitali J.A., Afb T., Linscheer G. Development of Quantitative Structure-Property Relationships for Screening of Tropodegradable Halocarbons. Proceedings of the Halon Options Technical Working Conference.

[32] Blowers P., Tetrault K.F., Trujillo-Morehead Y. (2008). Global Warming Potential Predictions for Hydrofluoroethers with Two Carbon Atoms. Theor. Chem. Acc..

[33] Wernet G., Hellweg S., Fischer U., Papadokonstantakis S., Hungerbühler K. (2008). Molecular-Structure-Based Models of Chemical Inventories Using Neural Networks. Environ. Sci. Technol..

[34] Wernet G., Papadokonstantakis S., Hellweg S., Hungerbühler K. (2009). Bridging Data Gaps in Environmental Assessments: Modeling Impacts of Fine and Basic Chemical Production. Green Chem..

[35] Kazakov A., McLinden M.O., Frenkel M. (2012). Computational Design of New Refrigerant Fluids Based on Environmental, Safety, and Thermodynamic Characteristics. Ind. Eng. Chem. Res..

[36] Fantke P., Bijster M., Hauschild M.Z., Huijbregts M., Jolliet O., Kounina A., Magaud V., Margni M., McKone T.E., Rosenbaum R.K. (2017). USEtox^®^ 2.0 Documentation, Version 1.1.

[37] Saouter E., Aschberger K., Fantke P., Hauschild M.Z., Bopp S.K., Kienzler A., Paini A., Pant R., Secchi M., Sala S. (2017). Improving Substance Information in USEtox^®^, Part 1: Discussion on Data and Approaches for Estimating Freshwater Ecotoxicity Effect Factors. Environ. Toxicol. Chem..

[38] Saouter E., Aschberger K., Fantke P., Hauschild M.Z., Kienzler A., Paini A., Pant R., Radovnikovic A., Secchi M., Sala S. (2017). Improving Substance Information in USEtox^®^, Part 2: Data for Estimating Fate and Ecosystem Exposure Factors. Environ. Toxicol. Chem..

[39] Song R., Keller A.A., Suh S. (2017). Rapid Life-Cycle Impact Screening Using Artificial Neural Networks. Environ. Sci. Technol..

[40] Zhu X., Ho C.-H., Wang X. (2020). Application of Life Cycle Assessment and Machine Learning for High-Throughput Screening of Green Chemical Substitutes. ACS Sustain. Chem. Eng..

[41] Rajapriya N., Kawajiri K. (2024). Deep Learning for GWP Prediction: A Framework Using PCA, Quantile Transformation, and Ensemble Modeling. arXiv.

[42] OECD (2004). The Report from the Expert Group on (Quantitative) Structure-Activity Relationships [(Q)SARs] on the Principles for the Validation of (Q)SARs.

[43] OECD (2014). Guidance Document on the Validation of (Quantitative) Structure-Activity Relationship [(Q)SAR] Models.

[44] OECD (2023). (Q)SAR Assessment Framework: Guidance for the Regulatory Assessment of (Quantitative) Structure—Activity Relationship Models, Predictions, and Results Based on Multiple Predictions.

[45] OECD (2024). (Q)SAR Assessment Framework: Guidance for the Regulatory Assessment of (Quantitative) Structure Activity Relationship Models and Predictions.

[46] European Parliament (2006). Regulation (EC) No 1907/2006 of the European Parliament and of the Council of 18 December 2006 Concerning the Registration, Evaluation, Authorisation and Restriction of Chemicals (REACH), Establishing a European Chemicals Agency, Amending Directive 1999/45/EC and Repealing Council Regulation (EEC) No 793/93 and Commission Regulation (EC) No 1488/94 as Well as Council Directive 76/769/EEC and Commission Directives 91/155/EEC, 93/67/EEC, 93/105/EC and 2000/21/EC.

[47] Mervin L.H., Johansson S., Semenova E., Giblin K.A., Engkvist O. (2021). Uncertainty Quantification in Drug Design. Drug Discov. Today.

[48] Williams A.J., Grulke C.M., Edwards J., McEachran A.D., Mansouri K., Baker N.C., Patlewicz G., Shah I., Wambaugh J.F., Judson R.S. (2017). The CompTox Chemistry Dashboard: A Community Data Resource for Environmental Chemistry. J. Cheminform..

[49] Kim S., Chen J., Cheng T., Gindulyte A., He J., Li Q., Shoemaker B., Thiessen P., Yu B., Zaslavsky L. (2023). Pubchem 2023 Update. Nucleic Acids Res..

[50] O’Boyle N.M., Banck M., James C.A., Morley C., Vandermeersch T., Hutchison G.R. (2011). Open Babel: An Open Chemical Toolbox. J. Cheminform..

[51] Moriwaki H., Tian Y.-S., Kawashita N., Takagi T. (2018). Mordred: A Molecular Descriptor Calculator. J. Cheminform..

[52] OECD (2007). ENV/JM/MONO(2007)2 2 OECD Environment Health and Safety Publications Series on Testing and Assessment No. 69 Guidance Document on the Validation of (Quantitative) Structure-Activity Relationship [(Q)SAR] Models.

[53] Chirico N., McLachlan M.S., Li Z., Papa E. (2024). In Silico Approaches for the Prediction of the Breakthrough of Organic Contaminants in Wastewater Treatment Plants. Environ. Sci. Process. Impacts.

[54] Rücker C., Rücker G., Meringer M. (2007). Y-Randomization and Its Variants in QSPR/QSAR. J. Chem. Inf. Model..

[55] Hastie T., Tibshirani R., Friedman J. (2009). The Elements of Statistical Learning. Data Mining, Inference, and Prediction.

[56] Gramatica P., Chirico N., Papa E., Cassani S., Kovarich S. (2013). QSARINS: A New Software for the Development, Analysis, and Validation of QSAR MLR Models. J. Comput. Chem..

[57] European Chemicals Agency (2025). How to Use and Report (Q)SARs -Practical Guide. August 2025.

[58] Sahigara F., Mansouri K., Ballabio D., Mauri A., Consonni V., Todeschini R. (2012). Comparison of Different Approaches to Define the Applicability Domain of QSAR Models. Molecules.

[59] Geng Y., Cao P., Feng N., Zhu Q., Qiu Y., Qi Z., Song Z. (2026). Machine Learning-Based Prediction of Global Warming and Ozone Depletion Potentials of Refrigerants. Chem. Eng. Sci..

[60] Chirico N., Sgariboldi A., Evangelista M., Papa E. (2026). QSAR-ME Profiler 2025: A New Software for QSA(P)R Predictions Supported by Structural Analysis. Chem. Res. Toxicol..

[61] Pinnock S., Hurley M.D., Shine K.P., Wallington T.J. (1995). Radiative Forcing of Climate by Hydrochlorofluorocarbons and Hydrofluorocarbons. J. Geophys. Res. Atmos..

[62] Reisinger A., Meinshausen M., Manning M., Bodeker G. (2010). Uncertainties of Global Warming Metrics: CO_2_ and CH_4_. Geophys. Res. Lett..

[63] Todeschini R., Consonni V. (2008). Handbook of Molecular Descriptors.

[64] Todeschini R., Consonni V. (2009). Molecular Descriptors for Chemoinformatics: Volume I: Alphabetical Listing/Volume II: Appendices, References.

[65] Halder A.K., Cordeiro M.N.D.S. (2019). Development of Multi-Target Chemometric Models for the Inhibition of Class I PI3K Enzyme Isoforms: A Case Study Using QSAR-Co Tool. Int. J. Mol. Sci..

[66] Zhao Q., Cui Y., Liu C., Wen H., Chen Y., Li P., Zhou Y., Wang Y., Jiang H., Wu Q. (2026). Machine Learning Model Based on Parallel Reaction Mechanisms for Predicting CO2 Capacity of Amine Solvents. Chem. Eng. Sci..

[67] Poola A.A., Prabhu P.S., Murthy T.P.K., Murahari M., Krishna S., Samantaray M., Ramaswamy A. (2023). Ligand-Based Pharmacophore Modeling and QSAR Approach to Identify Potential Dengue Protease Inhibitors. Front. Mol. Biosci..

[68] Hall L.H., Kier L.B. (1995). Electrotopological State Indices for Atom Types: A Novel Combination of Electronic, Topological, and Valence State Information. J. Chem. Inf. Comput. Sci..

[69] Zhang X., Kobayashi N., He M., Wang J. (2014). Radiative Efficiency Estimation of Organic Substance Based on Group Contribution Method. Energy Procedia.

[70] Hodnebrog Ø., Aamaas B., Fuglestvedt J.S., Marston G., Myhre G., Nielsen C.J., Sandstad M., Shine K.P., Wallington T.J. (2013). Global warming potentials and radiative efficiencies of halocarbons and related compounds: A comprehensive review. Geophys..

[71] Zhao G., Kim H., Yang C., Chung Y.G. (2024). Leveraging Machine Learning To Predict the Atmospheric Lifetime and the Global Warming Potential of SF_6_ Replacement Gases. J. Phys. Chem. A..

[72] Todeschini R., Ballabio D., Grisoni F. (2016). Beware of Unreliable *Q*^2^ ! A Comparative Study of Regression Metrics for Predictivity Assessment of QSAR Models. J. Chem. Inf. Model..

[73] Todeschini R. (1998). Introduzione alla chemiometria.

