# Supplementary material for: QSAR Models for Climate Impact Assessment: Born to Be Sustainable
Source: Toxics. 2026 Jul 28;14(8):667. doi: 10.3390/toxics14080667 (PMC13517518; doi:10.3390/toxics14080667)
Supplement: Supplementary file 1 [file toxics-14-00667-s001.zip › Supplementary Material File S3.pdf]

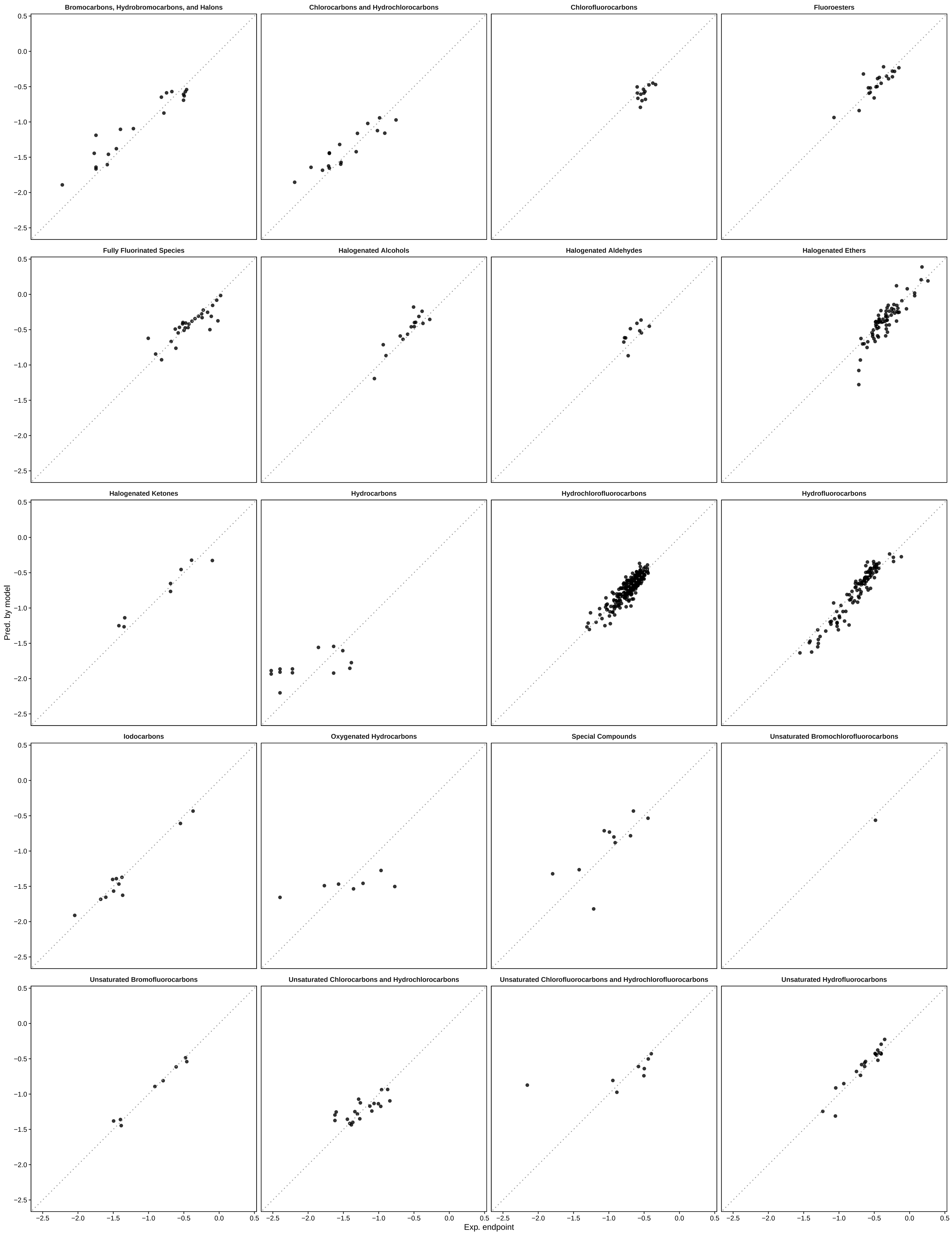
Bromocarbons, Hydrobromocarbons, and Halons
Chlorocarbons and Hydrochlorocarbons
Chlorofluorocarbons
Fluoroesters
Fully Fluorinated Species
Halogenated Alcohols
Halogenated Aldehydes
Halogenated Ethers
Halogenated Ketones
Hydrocarbons
Hydrochlorofluorocarbons
Hydrofluorocarbons
Iodocarbons
Oxygenated Hydrocarbons
Special Compounds
Unsaturated Bromochlorofluorocarbons
Unsaturated Bromofluorocarbons
Unsaturated Chlorocarbons and Hydrochlorocarbons
Unsaturated Chlorofluorocarbons and Hydrochlorofluorocarbons
Unsaturated Hydrofluorocarbons
Pred. by model
Exp. endpoint
0.5
0.0
-0.5
-1.0
-1.5
-2.0
-2.5
