# Supplementary material for: QSAR Models for Climate Impact Assessment: Born to Be Sustainable
Source: Toxics. 2026 Jul 28;14(8):667. doi: 10.3390/toxics14080667 (PMC13517518; doi:10.3390/toxics14080667)
Supplement: Supplementary file 1 [file toxics-14-00667-s001.zip › Supplementary Materials File S2.pdf]

Supplementary Materials S2

Comparative overview of selected representative studies (not exhaustive) 1
Metrics for the MLR-QSAR evaluation 3
Standardized residuals 4
Uncertainty 4
Frequency of classes in the dataset after curation. 5
Global Warming Potential–QSAR 5
Compound removed after data curation, divided by class 5
Bootstrap analysis 6
Outliers analysis 6
Bootstrap analysis, after elimination of outliers 8
Best GWP–QSAR 8
Outliers Analysis 12
Full model 16
Standardized coefficients 18
Radiative Efficiency–QSAR 19
Bootstrap analysis 19
BEST RE QSAR 20
Diagnostic Plot 20
Outliers analysis 22
Full model 25
Standardized coefficients 27
References 27

Comparative overview of selected representative studies (not exhaustive)

Table S1. Selected representative studies describing predictive approaches for estimating climate-related endpoints relevant to LCIA applications and chemical screening applications.

Ref.	Dataset	N° of substances	Endpoint	Methodology	Performance°
[1]	IPCC 2021 (mainly GHGs and some insulating gases)	165	GWP100yr	ML and GCM: ANN, RF, GBDT, SVM	R^2 test 0.82, MSE 0.015, RMSE 0.024 MAE 0.09

[2]	WMO 2018	495	GWP100yr	GCM and ML: ANN	Index of agreement training 0.99, testing 0.82
[3]	IPCC AR6	207 refrigerants and GHGs (single-component refrigerants)	GWP100 yr	Molecular descriptor-based modelling with quantile transformation preprocessing and dimensionality reduction strategies: FCNN.	RMSE 481.9 R^2 0.918
[4]		10 1,1-HFEs	GWP-20yr GWP-100yr GWP-500yr	Computational chemistry and thermodynamics approaches*	N.A.
[5]	Combining data from: IPCC 2007 and WMO 2010 and the literature	104	RE	New Semi empirical estimation (PM6 method) based on Pinnock approach [6], quantum-chemical calculation using Gaussian 09 package.	logRMSD 1.84
			GWP-100yr	From previous estimation of RE and atmospheric life time	logRMSD 3.0
[7]	IPCC 2007	29 chemicals form IPCC 2007	RE	GCM	Relative error % range 0 – 80.8
[8]	WMO, IPCC, EPA, work by [9]	235	RE	Computational chemistry: RE values computed using the values of the vibration frequencies and the associated intensities taken from B3LYP calculation.	MAE: 0.044 – 0.038, RMS: 0.075 – 0.079
[10]	IPCC AR6 report	196	GWP-100yr	Computational chemistry approach (e.g., DFT) with	R^2: 0.98, MAE: 0,22, RMSE= 0.30,

				ML: GBR, RF, SVR, KNN, LASSO, KRR, MLP	R^2_{EXT}: 0.90, MAE_{EXT}: 0,37, $RMSE_{EXT}$ = 0.48 (RF)
[11]	Ecoinvent v3.01	166	GWP-100yr	ML: ANN	Training: R^2 0.31, MRE 20%, validation test: R^2 0.21, MRE 88%, testing set: R^2 0.48, MRE 50%
[12]	WMO 2018	775	GWP-100yr	ML: Nonlinear model (i.e., RF, XGB, SVR and ANN) and linear models (i.e., MLR, LASSO and RR)	Training R^2 0.84 – 0.99 MAE 0.97 – 0.15 test: R2 0.74 -0.88 MAE 0.73-1.12

°Performance metrics reported as provided in the original studies.
**Combine prediction of radiative forcing values from density functional theory computations at B3LYP/631g level of theory.*
ML: machine learning; GCM: group contribution methods; ANN: artificial neurol network; GBR: gradient boosting regression; RF: random forest; DT: decision tree regression; KNN: k-nearest neighbours regression; SVR: support vector regression; ABR: adaBoost regressor; LASSO: least absolute shrinkage and selection operator; KRR: kernel ridge regression; MLP multilayer perception; FCNN: fully connected neural network; RMSE: root mean square error; R2: coefficient of determination; RMSD: root mean squared deviation; MAE: mean absolute error; RMS: root mean squared error; MRE: mean relative error; N.A.: not applicable (not reported in the study).

Metrics for the MLR-QSAR evaluation

Coefficient of determination (R^2):

$$R^2 = \frac{TSS - RSS}{TSS} = 1 - \frac{RSS}{TSS}$$

where

TSS is the total sum of squares: $TSS = \sum (y_i - \overline{y})^2$ and RSS stand for residual sum of squares $RSS = \sum_{i=1}^{n} (y_i - \hat{y})^2$;

Mean average error (MAE)

$$MAE = \frac{\sum_{i=1}^{n} |y_i - \hat{y}_i|}{n};$$

Leave-one-out cross-validated R^2 (Q^2_{LOO}):

$$Q^2_{LOO} = 1 - \frac{\sum_{i=1}^{n_{TR}} (y_i - \hat{y}_{i/i})^2}{\sum_{i=1}^{n_{TR}} (y_i - \bar{y})^2};$$

External validation metric Q^2_{F3} [13]:

$$Q_{F3}^2 = 1 - \frac{\frac{\sum_{i=1}^{n_{TEST}} (y_i - \hat{y}_i)^2}{n_{TEST}}}{\frac{\sum_{i=1}^{n_{TR}} (y_i - \bar{y})^2}{n_{TR}}}$$

where

$\bar{y}$: average of the experimental responses;

y_i: experimental value of the i-th chemical;

$\hat{y}_i$: predicted value of the i-th chemical;

n: number of chemicals in either the training or test set, unless otherwise specified, according to which the metric refers to;

$\hat{y}_{i/i}$: predicted value for the i-th chemical when removed from the model, calculated without the i-th chemical.

Standardized residuals

$$r_i' = \frac{r_i}{s \times \sqrt{1 - h_{ii}}}$$

where, following the method described in Todeschini's book " Introduction of Chemiometrics" [14],

h_{ii} : diagonal elements of the HAT influence matrix (Equation 1 in the article);

$s = \sqrt{\frac{RSS}{n - p'}}$: standard error of the estimate where n is the number of compounds in the training set and p' total number of parameters in the regression model;

$r_i = y_i - \hat{y}_i$: ordinary residues;

y_i: experimental value of the i-th chemical;

$\hat{y}_i$: predicted value of the i-th chemical.

Uncertainty

Prediction interval:

Prediction uncertainty (i.e., prediction interval) is calculated as

$$uncert = \pm t_{stud\ \alpha/2} \times s \times \sqrt{1 + HAT_i}$$

where

$t_{stud\ \alpha/2}$: t-student calculated for $\alpha/2$ = 0.025.

Mordred Descriptor Name	AETA_eta_L	ETA_epsilon_5	JGT10

Table S2. Conversion table showing the differences in nomenclature between the table between Mordred descriptors [51] and those in QSAR-ME Profiler 2025 [59]..

Frequency of classes in the dataset after curation.

Class	n° of compound	% of compound
Hydrochlorofluorocarbons	264	39%
Hydrofluorocarbons	107	16%
Halogenated Ethers	71	11%
Fully Fluorinated Species	28	4%
Unsaturated Hydrofluorocarbons	20	3%
Unsaturated Chlorocarbons and Hydrochlorocarbons	20	3%
Fluoroesters	20	3%
Bromocarbons, Hydrobromocarbons, and Halons	19	3%
Chlorocarbons and Hydrochlorocarbons	17	3%
Halogenated Alcohols	15	2%
Hydrocarbons	13	2%
Chlorofluorocarbons	13	2%
Iodocarbons	11	2%
Halogenated Aldehydes	10	1%
Special Compounds	10	1%
Unsaturated Chlorofluorocarbons and Hydrochlorofluorocarbons	8	1%
Unsaturated Bromofluorocarbons	8	1%
Halogenated Ketones	8	1%
Oxygenated Hydrocarbons	7	1%
No Details About Class	2	0%
Unsaturated Bromochlorofluorocarbons	1	0%

Table S3. Frequency in terms of number and percentage of the class of substances present in the dataset after curation, reported in the supplementary materials S1 (Sheet 01). The total number of compounds is 672.

Global Warming Potential–QSAR

Compound removed after data curation, divided by class

Class	N° of compound with value <1	N° of compound with value <<1	N° TOTAL
**Hydrocarbons**	1	12	13
**Oxygenated Hydrocarbons**	1	5	6
**Hydrofluorocarbons**	4		4
**Unsaturated Hydrofluorocarbons**	8	7	15

**Chlorocarbons and Hydrochlorocarbons**	4	3	7
**Unsaturated Chlorocarbons and Hydrochlorocarbons**	7	10	17
**Unsaturated Chlorofluorocarbons and Hydrochlorofluorocarbons**	2	3	5
**Bromocarbons, Hydrobromocarbons, and Halons**	3	2	5
**Unsaturated Bromofluorocarbons**	3	5	8
**Unsaturated Bromochlorofluorocarbons**		1	1
**Fully Fluorinated Species**	2	3	5
**Halogenated Ethers***	1	2	4
**Fluoroesters**		2	2
**Halogenated Alcohols**	6	1	7
**Halogenated Ketones**	2	4	6
**Halogenated Aldehydes**	3	7	10
**Iodocarbons**	1	10	11
**Special Compounds**	3	3	6

Table S4. Table reporting the quantities and the reasons why certain substances were excluded from the initial dataset of GWP endpoint, organized by substance class, to define the final GWP dataset, reported in Supplementary Materials S1_Sheet 02.

* Compound HFE-236fa has a value of 1.30 and is not consistent with the other data, so it has been eliminated. It is inconsistent because the decimals are expressed with a "." and the ", " is used for thousands.

Bootstrap analysis

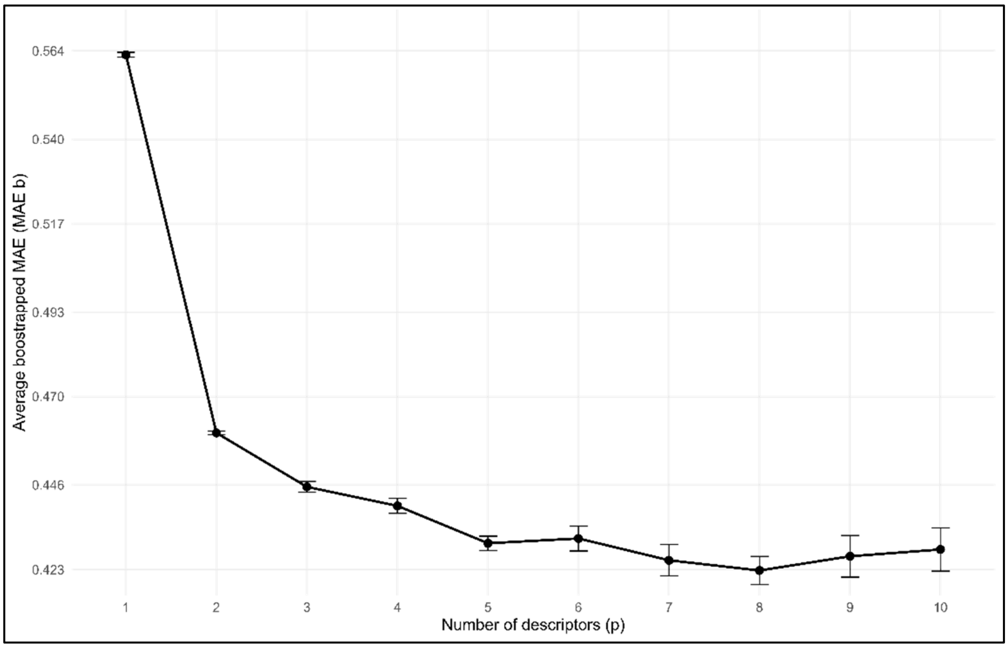

p	SE	MAE b
1	0.001	0.563
2	0.001	0.460
3	0.001	0.445
4	0.002	0.440
5	0.002	0.430
6	0.003	0.431
7	0.004	0.426
8	0.004	0.423
9	0.006	0.427
10	0.006	0.428

Figure S1. Bootstrap analysis of the GWP- MLR models.

Outliers analysis

ID_GWP	Outliers_Freq.*	Chemical name	Class	LogGWP

**379**	25	1-Fluorohexane	Hydrofluorocarbons	2.859
**385**	25	(Z)-HFO-1336mzz	Unsaturated Hydrofluorocarbons	0.301
**386**	25	HFO-1447fz	Unsaturated Hydrofluorocarbons	0.477
**388**	25	(E)-HFO-1438ezy	Unsaturated Hydrofluorocarbons	0.477
**438**	25	(E)-Perfluoro-2-butene	Fully Fluorinated Species	0
**457**	25	HFE-263mf	Halogenated Ethers	0.301
**479**	25	1,1,2,2-tetrafluoro-3-methoxy-propane	Halogenated Ethers	0.301
**517**	25	Perfluorobutyl acetate	Fluoroesters	0.301
**518**	25	Perfluoropropyl acetate	Fluoroesters	0.301
**475**	23	HFE-356mmz1	Halogenated Ethers	0.954

*Table S5. Substances considered as outliers for the dataset GWP. * Number of models for which the compound is defined as a potential outliers.*

Compounds with ID_GWP 457, 479, and 475 belong to the halogenated ethers class, which is represented in the training set by 46 substances, with an average experimental GWP value of 1812, a maximum of 14100, and a minimum of 0.30. Among these, 36 compounds have GWP values greater than 100 and 15 greater than 1000. Even if the three compounds do not present substantial structural differences from other chemicals in the same class, they have markedly lower GWP values, namely 0.30, 0.30, and 0.95, respectively. In the absence of other evidence which justifies these low GWP values these data have been considered as inaccurate and eliminated.

Compounds with ID_GWP 385, 386, and 388 belong to the unsaturated hydrofluorocarbons class, which is represented in the training set by only five substances. This class exhibits an average experimental GWP value in the training set of 14.8, with a maximum of 54 and a minimum of 2. The removed substances display values of 2, 3, and 3 and, unlike the other class members, are characterized by non-cyclic structures, suggesting a structural origin for their anomalous behaviour, i.e., the chemical class is underrepresented within the dataset and the model is not sensitive to structural diversity within the small unsaturated hydrofluorocarbons class.

The compound with ID_GWP 379 belongs to the hydrofluorocarbons class, represented in the training set by 80 substances. In this case, the experimental GWP value is not anomalous with respect to the class distribution; however, the molecule is structurally distinct due to a high number of hydrogen atoms (H=13).

Finally, the compound with ID_GWP 438, belonging to the fully fluorinated species class, exhibits an experimental GWP value of 1, whereas the other compounds in the same class show substantially higher values. Specifically, the 17 training-set substances in this class have an average GWP value of 9819.64, with a minimum of 1 and a maximum of 32300. Despite this, in the PCA space, ID_GWP 438 is located among compounds characterized by high experimental GWP values. Also in this case, in the absence of other evidence which justifies this low GWP value, it was considered inaccurate and eliminated.

Bootstrap analysis, after elimination of outliers

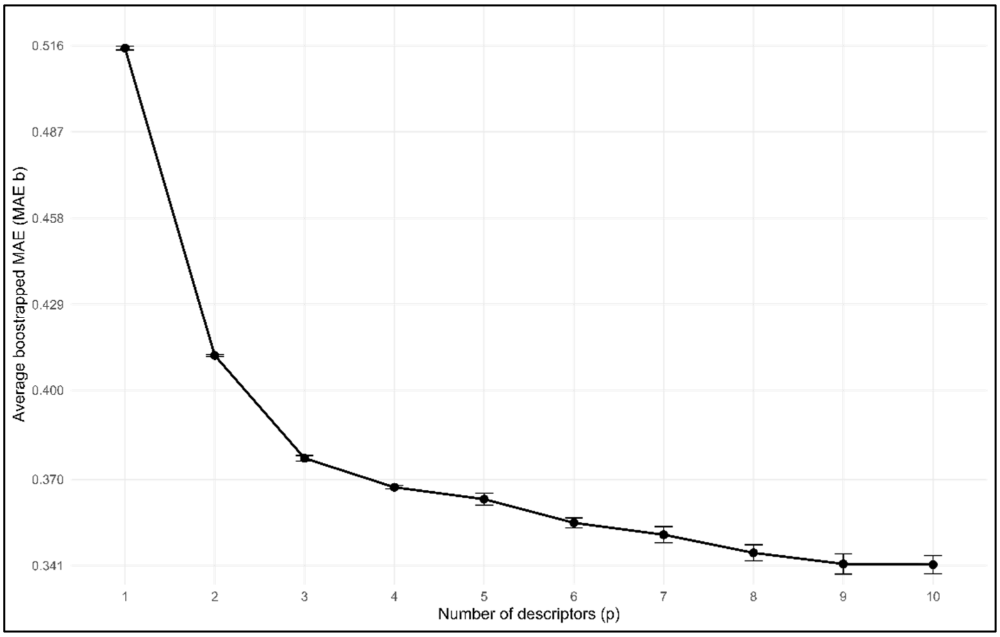

p	SE	MAE b
1	0.001	0.515
2	3.58E-04	0.412
3	0.001	0.377
4	0.001	0.367
5	0.002	0.363
6	0.002	0.355
7	0.003	0.351
8	0.003	0.345
9	0.003	0.342
10	0.003	0.341

Figure S2. Bootstrap analysis of the GWP- MLR models (after the elimination of outliers).

Best GWP–QSAR

Diagnostic plots

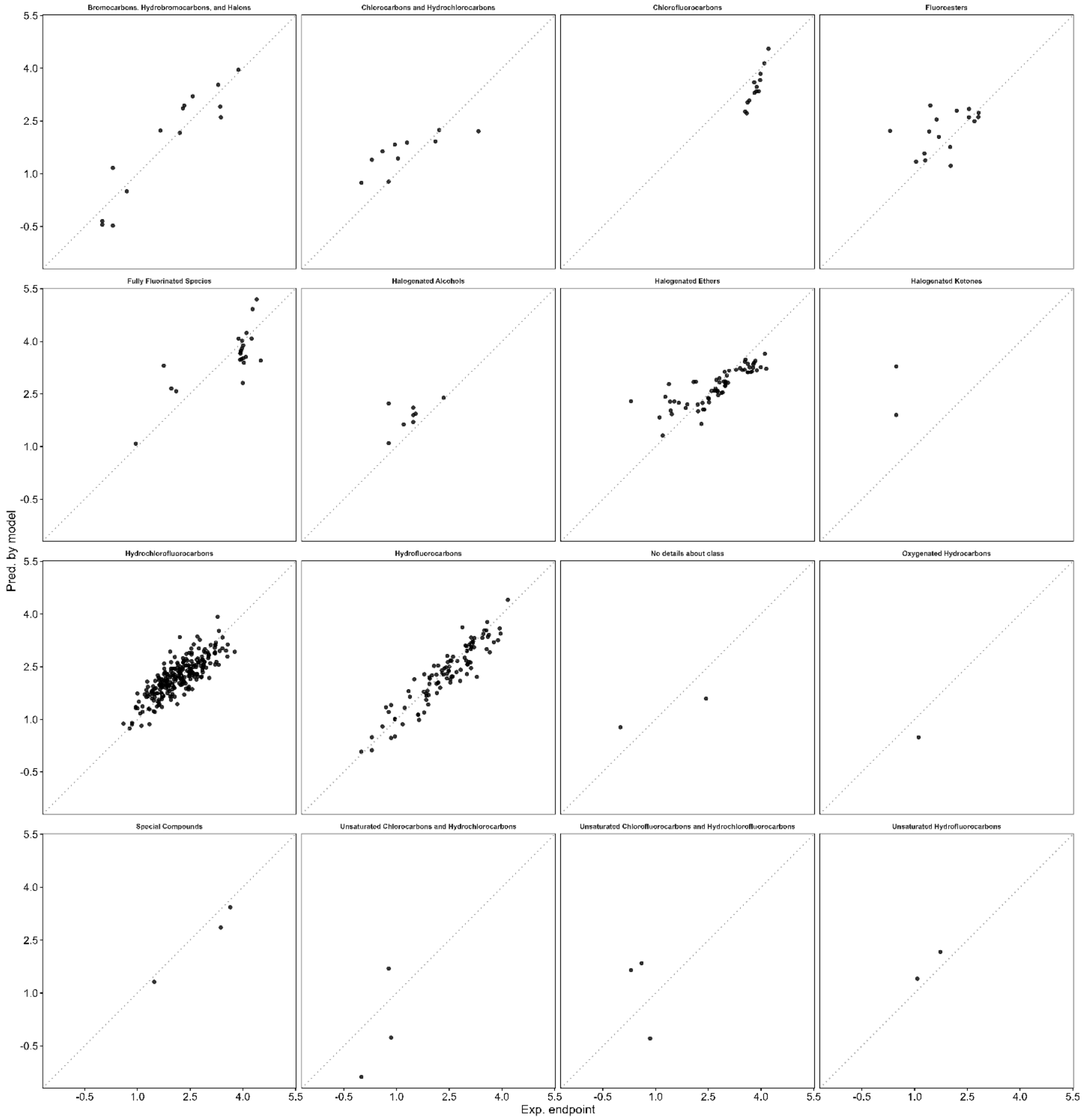

Figure S3. Regression diagnostic plots for the MLR-QSAR model: comparison between experimental (Exp. endpoint) and model predicted (Pred. by model) values for GWP endpoint. Data are divided by chemical class and displayed in separate panels.

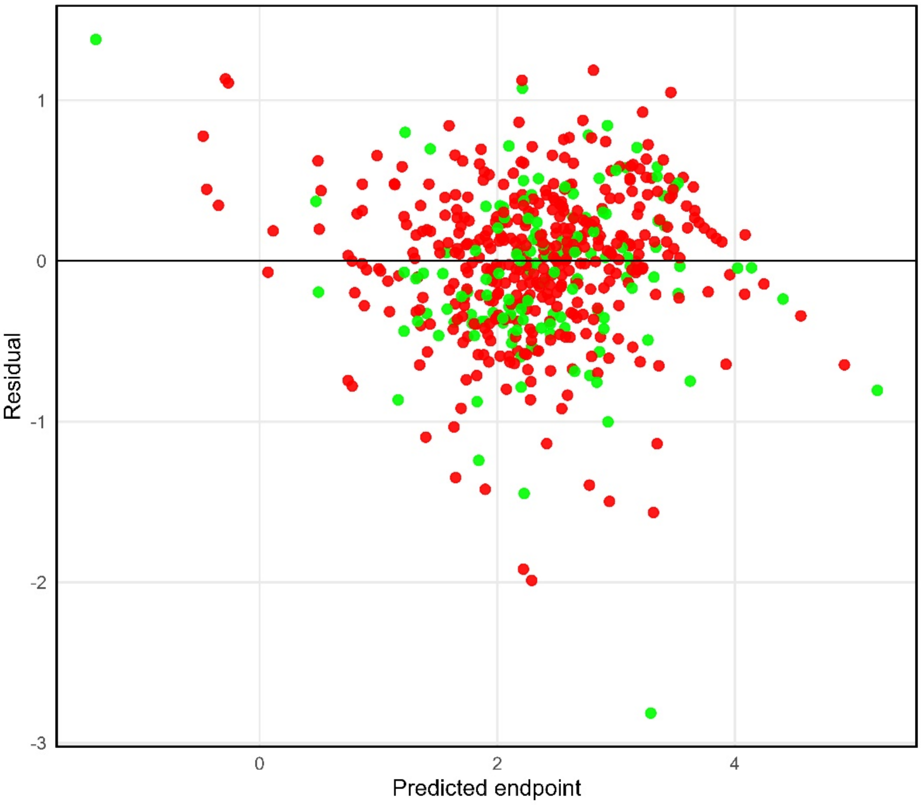

Figure S4. Plot of the residuals. Red points represent the training-set substances, while green points represent the test-set substances.

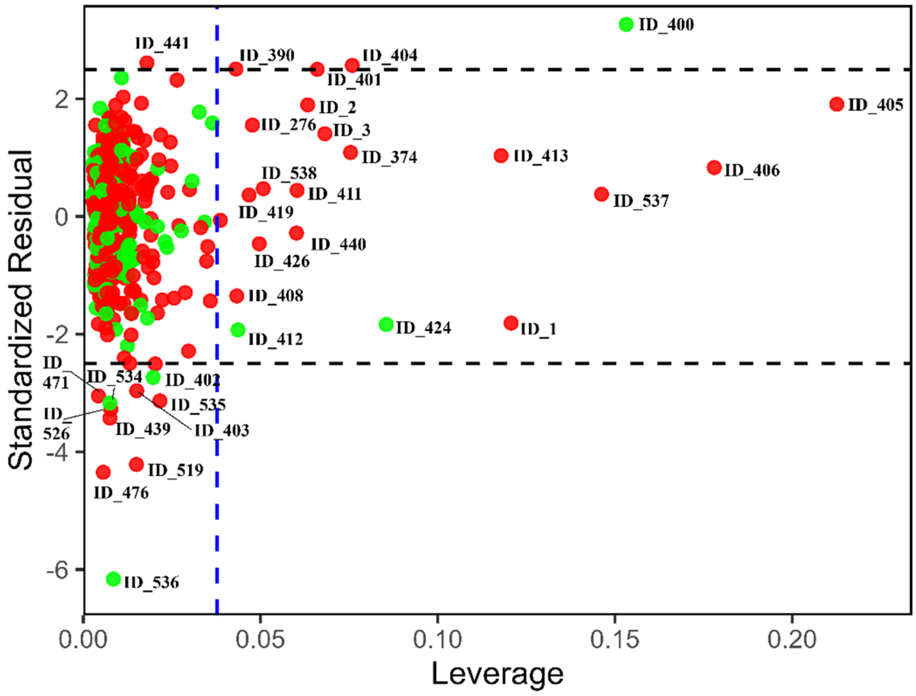

Figure S5. Williams plot of the split MLR-QSAR model of the GWP endpoint, showing standardized residuals as a function of leverage for the training (red) and test (green) sets. The horizontal dashed lines indicate the ±2.5 standardized residual thresholds used to identify response outliers, while the vertical blue dashed line represents the leverage threshold (h=0.038), defining the applicability domain of the model. The corresponding name of highlight chemicals is provided in Table S6.*

ID_GWP	Name of Chemical	Outliers and/or Outside the structural domain
1	Carbon dioxide	Outside the structural applicability domain
2	Nitrous oxide	Outside the structural applicability domain
3	Methyl formate	Outside the structural applicability domain
276	HCFC-271ba	Outside the structural applicability domain
374	HFC-b-3-10-1sy	Outside the structural applicability domain
389	Methyl chloroform	Outside the structural applicability domain
390	Carbon tetrachloride	Outlier and outside the structural applicability domain
400	1,4-dichlorobenzene	Outlier and outside the structural applicability domain
401	1,2-dichloro-3-(trichloromethyl)-benzene (DCTCB)	Outside the structural applicability domain
404	p-chlorobenzotrifluoride	Outlier and outside the structural applicability domain
405	Methyl bromide	Outside the structural applicability domain
406	Methylene bromide	Outside the structural applicability domain
408	Halon-1202	Outside the structural applicability domain
411	Bromochloromethane	Outside the structural applicability domain
412	Bromodichloromethane	Outside the structural applicability domain
413	1,2-dibromoethane	Outside the structural applicability domain
419	Nitrogen trifluoride	Outside the structural applicability domain
424	Sulfur hexafluoride	Outside the structural applicability domain
426	PFC-14 (Perfluoromethane)	Outside the structural applicability domain
440	Hexafluorobenzene	Outside the structural applicability domain
537	Carbonyl Sulfide	Outside the structural applicability domain
538	Sulfuryl fluoride	Outside the structural applicability domain
17	HCFC-21	Outlier
402	HCFO-1233xf (2-chloro-3,3,3-fluoro-1-propene)	Outlier
403	1,2-dichloro-3,3,3-trifluoro-propene (Z)	Outlier
439	Perfluoro(2-methyl-2-pentene)	Outlier
441	PFPHP- Perfluoroperhydrophen-anthrene (Vitreon, Flutec PP 11)	Outlier
471	HFE-356mff2	Outlier
476	HFE-365mcf3	Outlier
519	Perfluoroethyl acetate	Outlier
526	Difluoromethyl 2,2,2-trifluoro-acetate	Outlier
534	2,2-3,3,4,4,5,5,5-fluoro-1-pentanol	Outlier
535	1,1,1-trifluoropropan-2-one	Outlier
536	1,1,1,3,3,3-hexafluoropropan-2-one	Outlier

Table S6. Corresponding ID and Name of outliers and/or chemicals outside the structural domain of MLR-QSAR model of the GWP endpoint.

Outliers Analysis

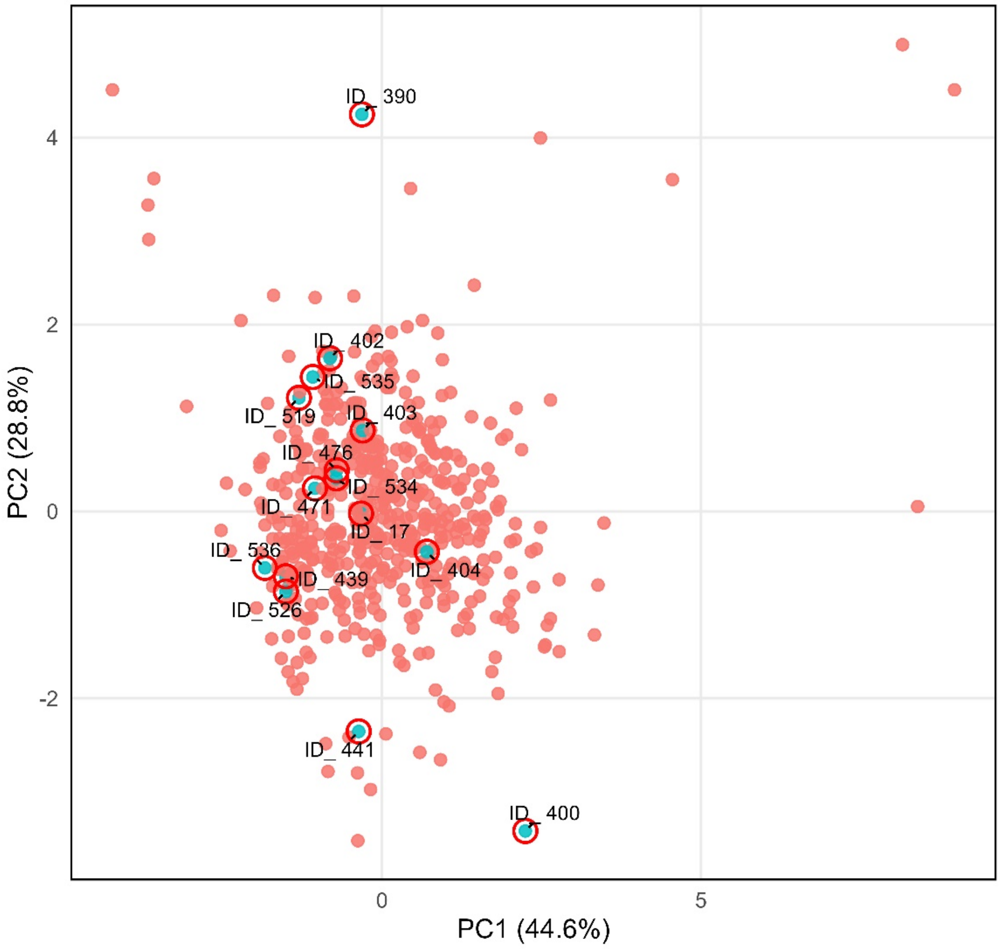

Figure S6. Plot of PC1 vs PC2 from the PCA of the GWP model dataset with loadings (molecular descriptor of the model). The blue light points represent the scores of the outliers, i.e., observations with standardized residuals beyond the cut-off (greater than 2.5 or less than –2.5), while the pink points represent those within the cut-off limits.

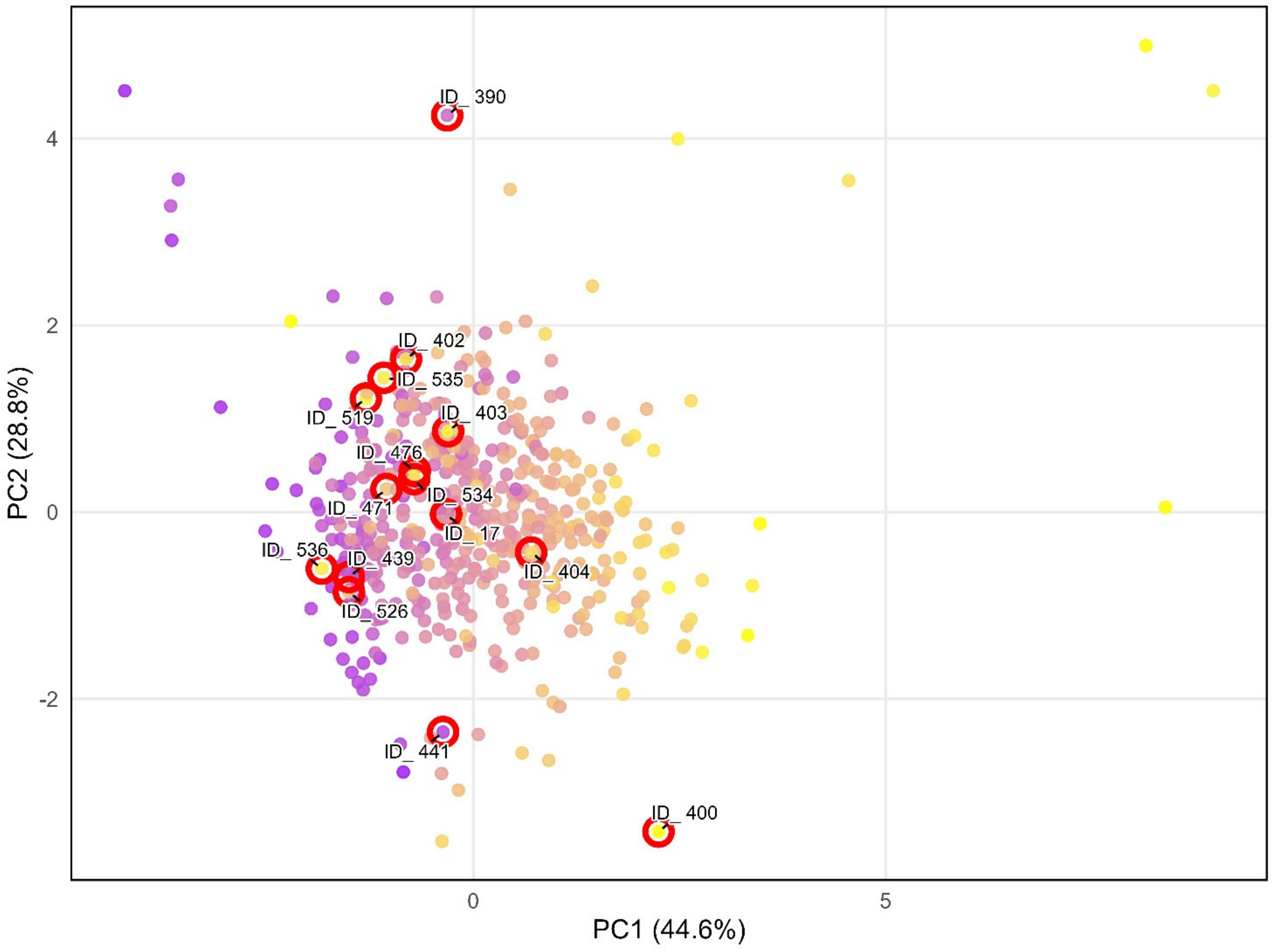

Figure S7. PC1 vs PC2 plot from the PCA of the GWP model dataset, shown without loadings (the molecular descriptors) to improve visual clarity. The score points are colored with a yellow-to-blue gradient reflecting the experimental GWP values (yellow = low GWP, purple = high GWP). The points marked with a red circle correspond to those identified as outliers in the previous plot , as their standardized residuals fall outside the cut-off values.

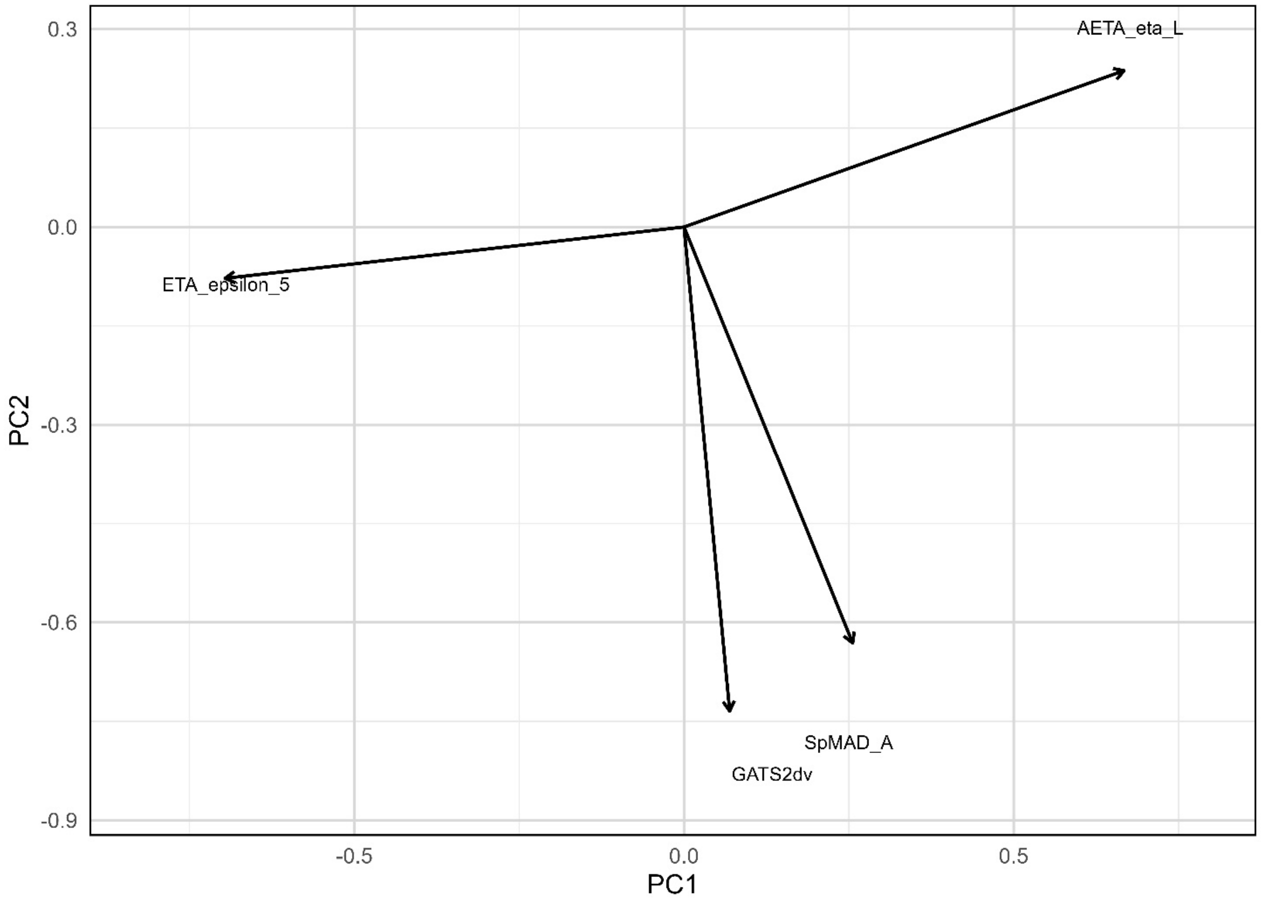

Figure S8. Loadings of the selected variables on the first two principal components (PC1 and PC2).

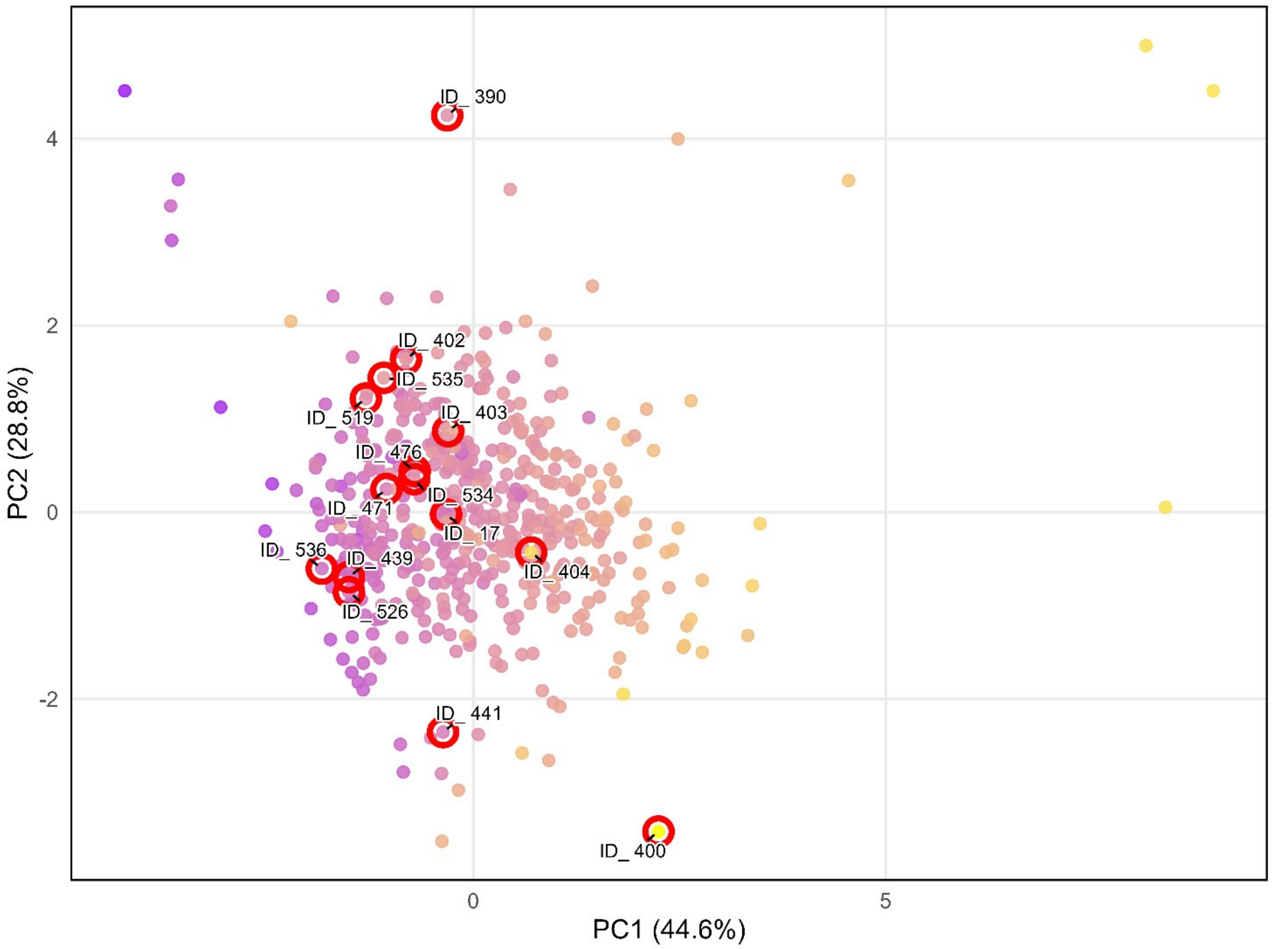

Figure S9. PC1 vs PC2 plot from the PCA of the GWP model dataset, shown without loadings (the molecular descriptors) to improve visual clarity. The score points are colored with a yellow-to-purple gradient reflecting the predicted GWP values (yellow = low GWP, purple = high GWP).

Additional analysis of response outliers:

The compound ID_GWP 390 belongs to the chlorocarbons and hydrochlorocarbons class and exhibits an experimental GWP value significantly higher than the average of its class (Average $logGWP_{100\text{-}yr}$ = 1.26, $logGWP_{100\text{-}yr}$ of ID_GWP 390 = 3.33). As shown in Figure S9, its compound is predicted similarity to its nearest neighbours in the PCA space, resulting in an underestimation of its climate impact. The compound ID_GWP 400, belonging to the unsaturated chlorocarbons and hydrochlorocarbons, is part of the test set. This class is represented in the training set by only two compounds, one of which (ID_GWP 401) is also outside the structural applicability domain. Both compounds exhibit low experimental GWP values and are overestimated by the model, a behaviour consistent with the documented difficulty in accurate prediction of low-GWP substances in the literature [2].

The remaining response outliers are distributed within the PCA space among compounds characterized by medium- and high-GWP values, despite having low experimental GWP. Therefore, they are predicted similarly to their neighbours (Figures S9), indicating that the descriptors of the model may not be sufficiently sensitive to the structural differences that lead to reduced GWP values.

The outliers ID_GWP 402 (test set), ID_GWP 403, and ID_GWP 404 (training set) belong to the unsaturated chlorofluorocarbons and hydrochlorofluorocarbons, which are entirely represented by these three compounds in the dataset. Their standardized residuals are close to the cut-off threshold, with values of -2.73, -2.96 and 2.57, respectively. In the PCA space, these compounds are positioned among the hydroclorofluorocarbons, explaining their predicted behaviour.

The compound ID_GWP 439, belonging to the fully fluorinated species class, is located in PCA space close to halogenated ethers. Its molecular descriptor values are similar to compounds characterized by $logGWP_{100\text{-}yr}$ values between 3 and 4, whereas its experimental $logGWP_{100\text{-}yr}$ is substantially lower (1.75).

Within the halogenated ethers class, two additional response outliers were identified (ID_GWP 471 and ID_GWP 476). Both compounds exhibit low experimental $logGWP_{100\text{-}yr}$ values but are overestimated by the model, as their descriptor values closely resemble those of compounds with higher GWP.

Finally, the outliers ID_GWP 519 and ID_GWP 526, belonging to the fluoroesters class, are part of the training set and exhibit low experimental $logGWP_{100\text{-}yr}$ (0.30 and 1.44, respectively). The average experimental $logGWP_{100\text{-}yr}$ for this class is 1.86. As shown in Figure S9, these compounds are positioned in the PCA space among substances with higher experimental GWP, again indicating insufficient model sensitivity to the features responsible for their lower climate impact. A similar behaviour is observed for ID_GWP 535 and 536 (halogenated ketones) and ID_GWP 534 (halogenated alcohol).

Full model

$$logGWP_{100\text{-}yr} = -3.16\ (\pm 1.18) + 6.16\ (\pm 0.50) \times ETA\_epsilon\_5 - 3.85(\pm 0.61) \times SpMAD\_A + 0.77(\pm 0.15) \times GATS2dv + 2.16(\pm 1.02) \times AETA\_eta\_L$$

N°: 529 R^2: 0.73, MAE_{tr}: 0.35, Q^2_{loo}: 0.72, R^2_{Yscr}: 0.01

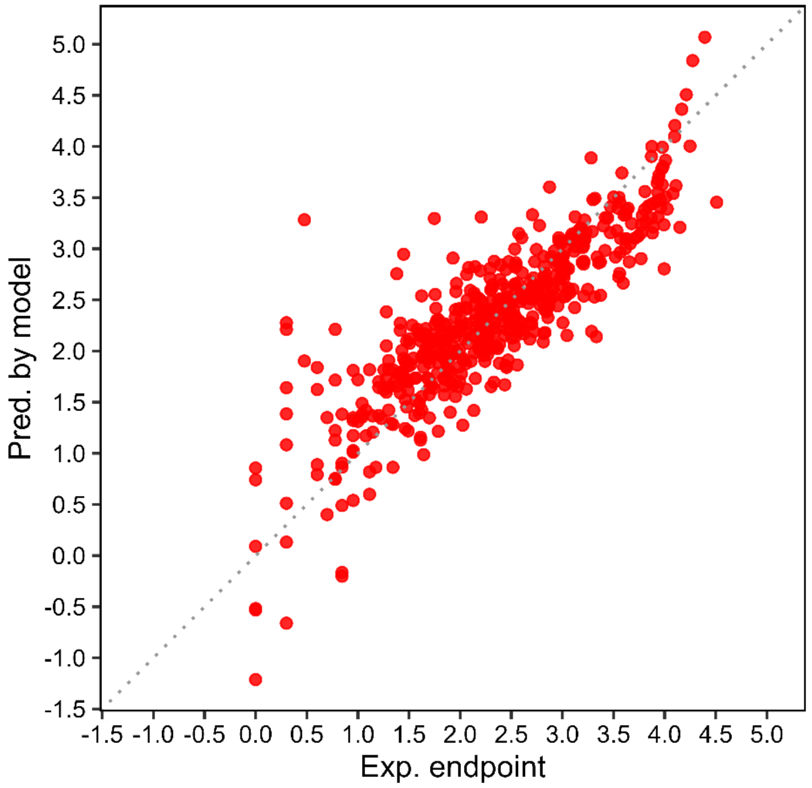

Figure S10. Regression diagnostic plots for the full MLR-QSAR model: comparison between experimental (Exp. endpoint) and model predicted (Pred. by model) values for GWP endpoint.

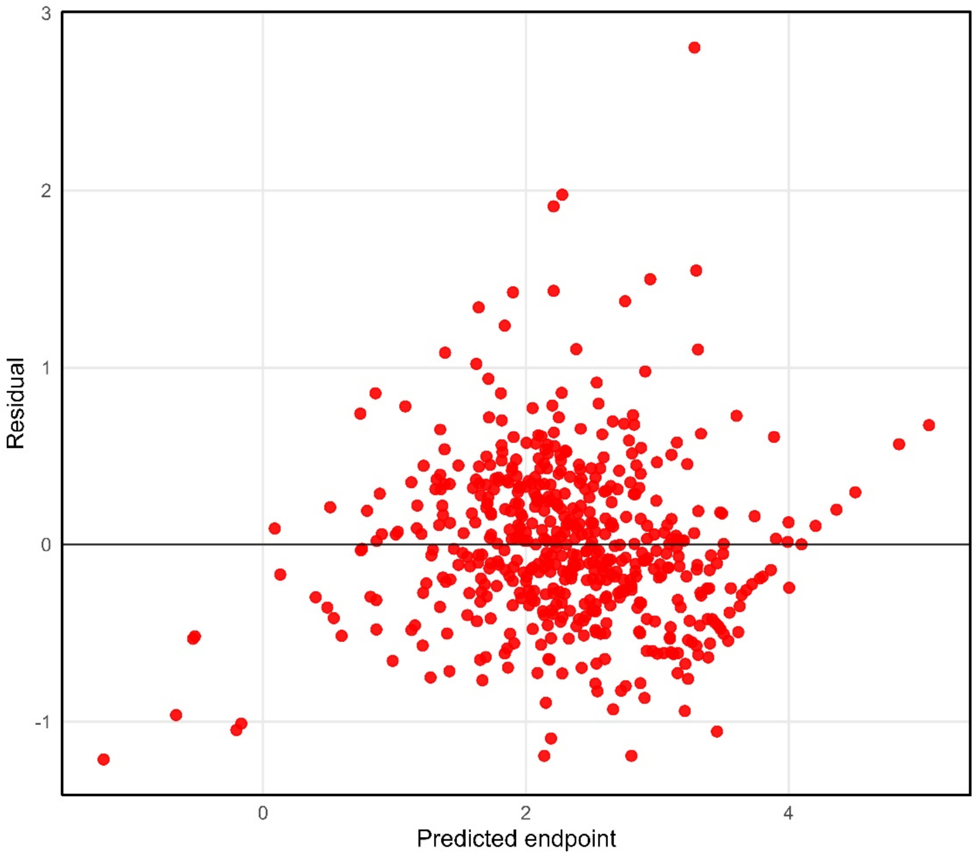

Figure S11. Plot of the residuals.

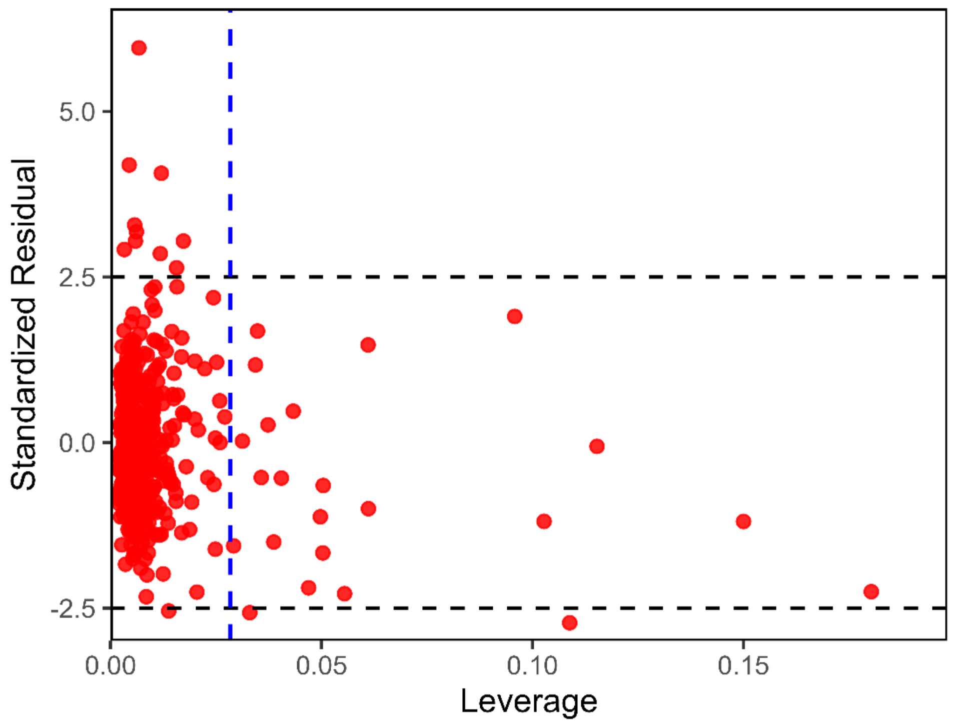

Figure S12. Williams plot of the full MLR-QSAR model of the GWP endpoint, showing standardized residuals as a function of leverage. The horizontal dashed lines indicate the ±2.5 standardized residual thresholds used to identify response outliers, while the vertical blue dashed line represents the leverage threshold (h= 0.0284), defining the applicability domain of the model.*

Standardized coefficients

TableS7. Standardized coefficients of the GWP model equations obtained for the split and full datasets.

GWP Model	ETA_epsilon_5	SpMAD_A	GATS2dv	AETA_eta_L
Split model	0.8836	-0.2985	0.226	0.185
Full model	0.829	-0.2933	0.2276	0.1395

Radiative Efficiency–QSAR

Bootstrap analysis

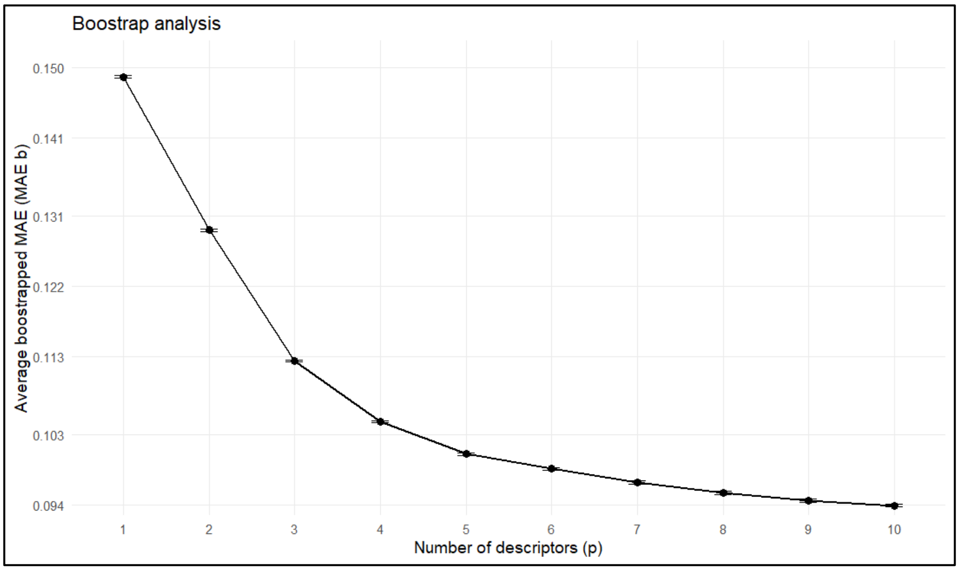

p	SE	MAE b
1	1.88E-04	0.149
2	1.64E-04	0.129
3	1.54E-04	0.112
4	1.27E-04	0.105
5	1.34E-04	0.101
6	1.70E-04	0.099
7	1.74E-04	0.097
8	2.20E-04	0.096
9	1.97E-04	0.095
10	1.86E-04	0.094

Figure S13. Bootstrap analysis of the RE-QSAR.

BEST RE QSAR

Diagnostic Plot

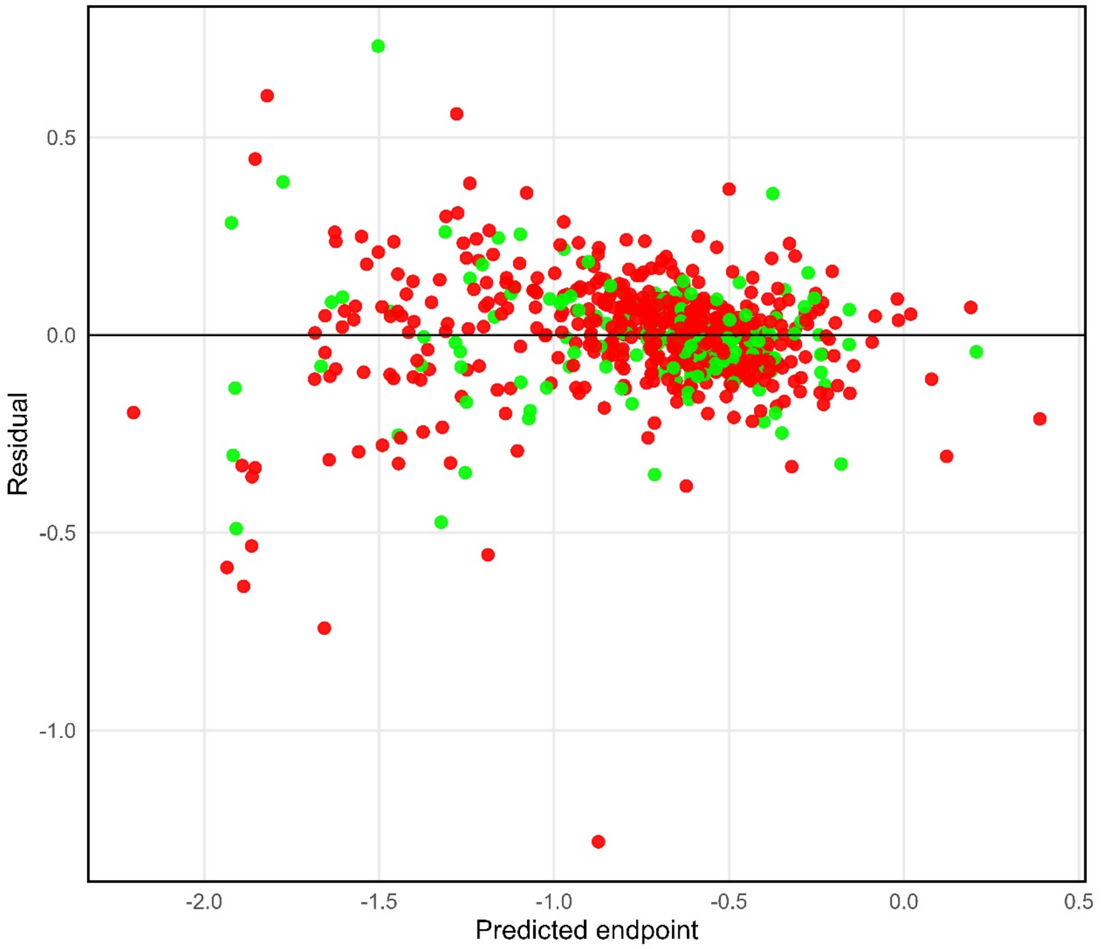

Figure S14. Plot of the residuals. Red points represent the training-set substances, while green points represent the test-set substances.

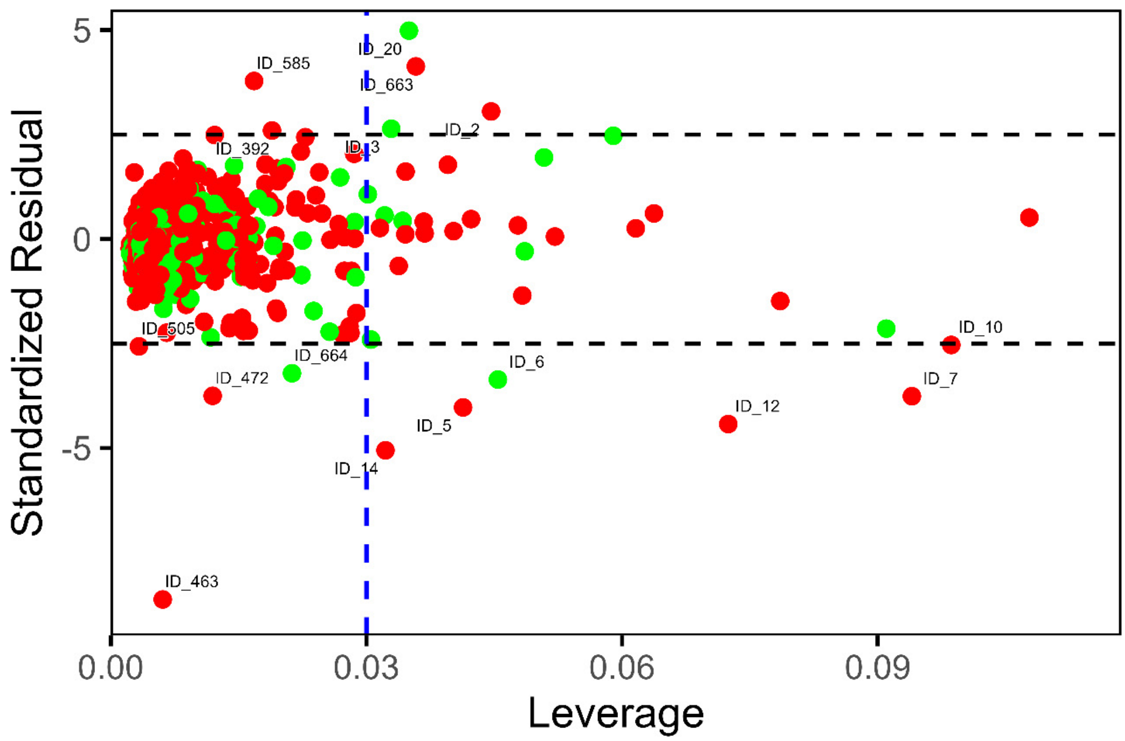

Figure S15. Williams plot of the split MLR-QSAR model. Red points represent the training-set substances, while green points represent the test-set substances. The horizontal dashed lines indicate the ±2.5 standardized residual thresholds used to identify response outliers, while the vertical blue dashed line represents the leverage threshold (h= 0.0298), defining the applicability domain of the model. The corresponding name of highlight chemicals is provided in Table S8.*

ID_RE	Name of chemical
2	Ethene
3	Acetylene
5	Propane, R-290
6	n-butane
7	Isobutane, R-600a
10	Isopentane
12	Benzene
14	Formaldehyde
20	Methylal
392	HFC-b-392pz
463	1,1-dichloro-2,2-difluoro-ethene
472	Bromoform
505	PFC-14 (Perfluoromethane)
585	Fluoro(methoxy)methane
663	Ammonia
664	Carbonyl Sulfide

Table S8. Corresponding ID and Name of outliers and/or chemicals outside the structural domain of MLR-QSAR model of the RE endpoint.

Outliers analysis

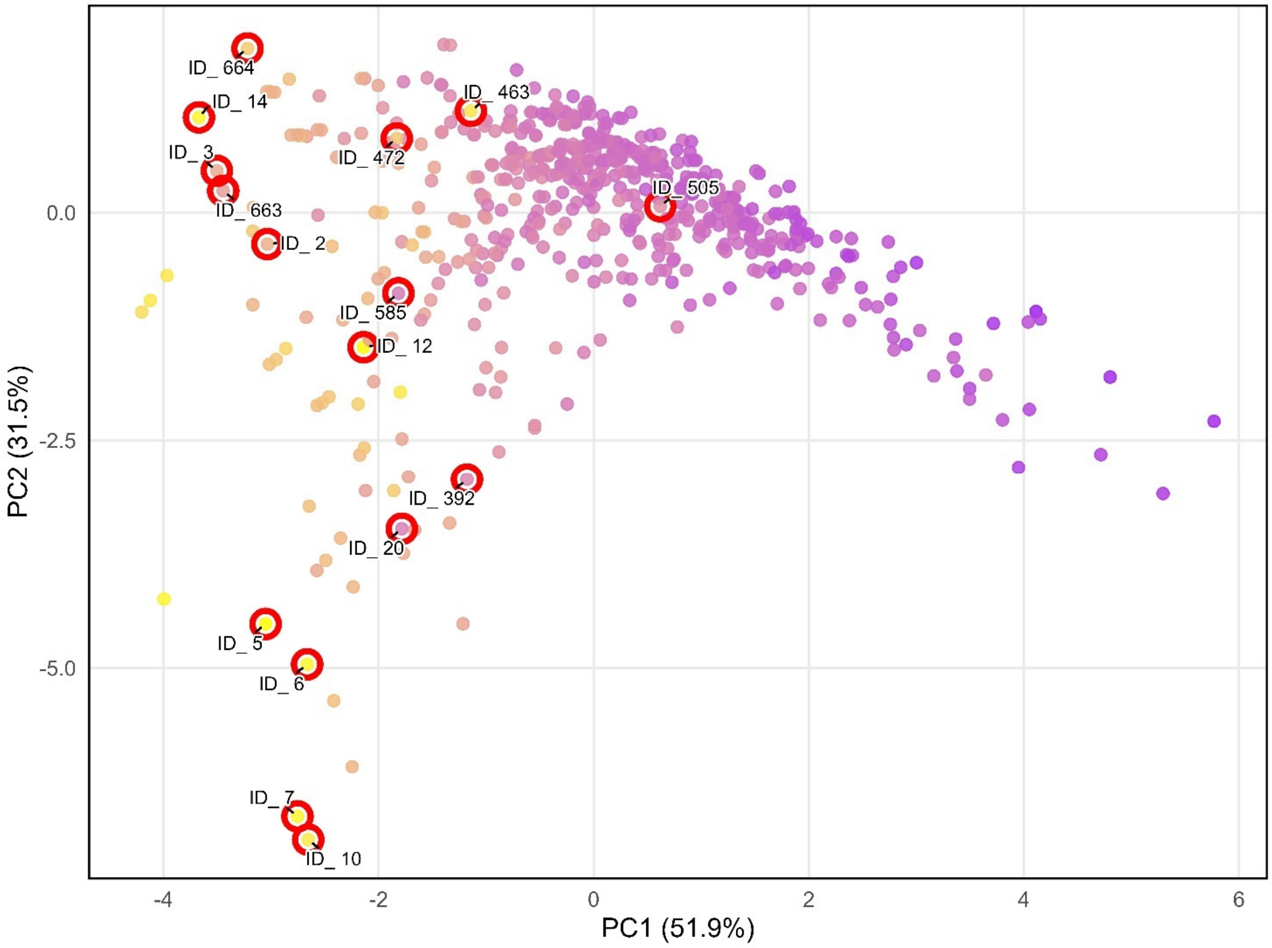

Figure S16. PC1 vs PC2 plot from the PCA of the RE model dataset, shown without loadings to improve visual clarity. The score points are colored with a yellow-to-purple gradient reflecting the experimental RE values (yellow = low RE, purple = high RE). The points marked with a red circle correspond to those identified as outliers in the previous plot , as their standardized residuals fall outside the cut-off values.

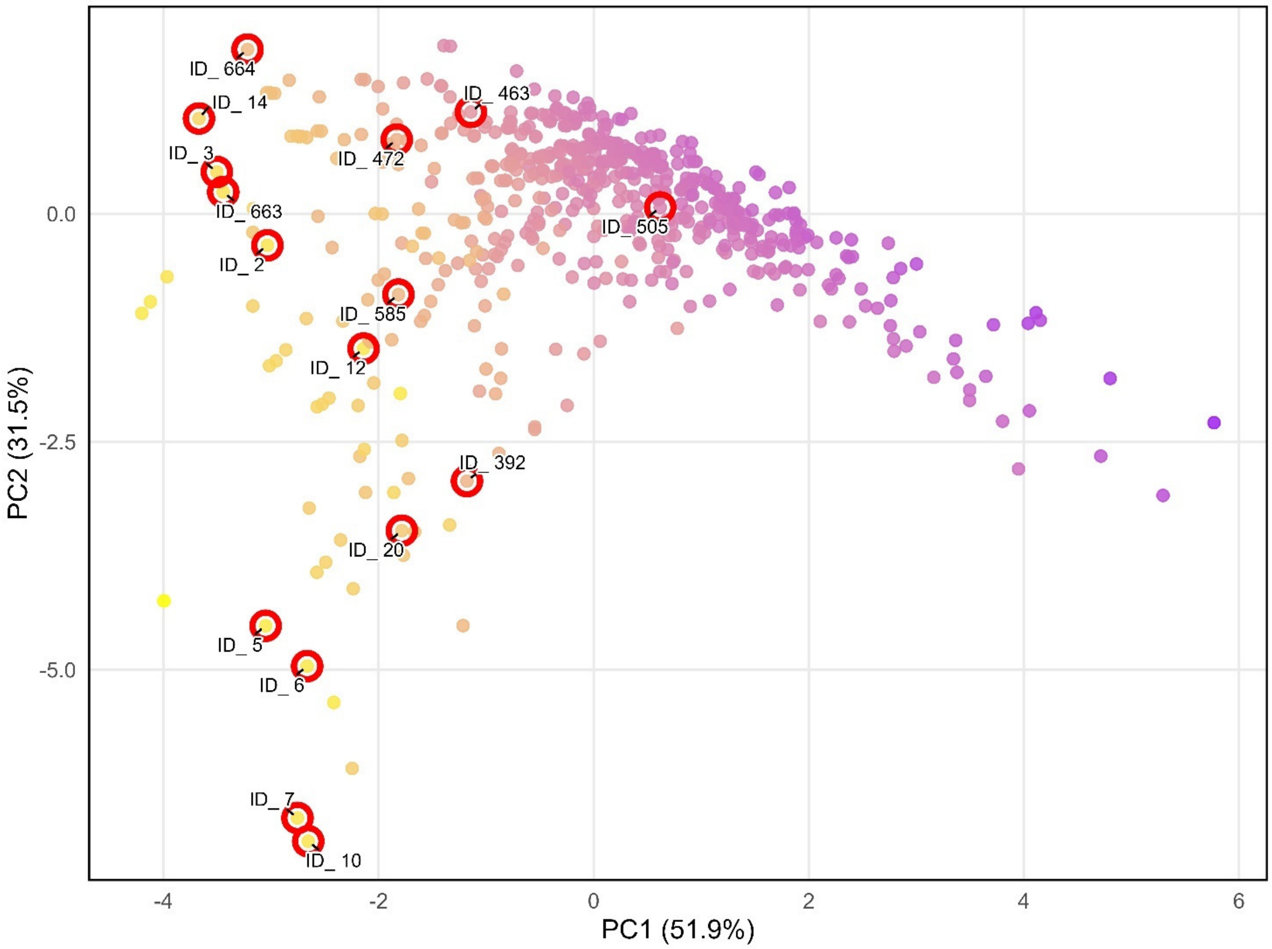

Figure S17. PC1 vs PC2 plot from the PCA of the RE model dataset, shown without loadings to improve visual clarity. The score points are colored with a yellow-to-purple gradient reflecting the predicted RE values (yellow = low RE, purple = high RE). The points marked with a red circle correspond to those identified as outliers in the previous plot,as their standardized residuals fall outside the cut-off values.

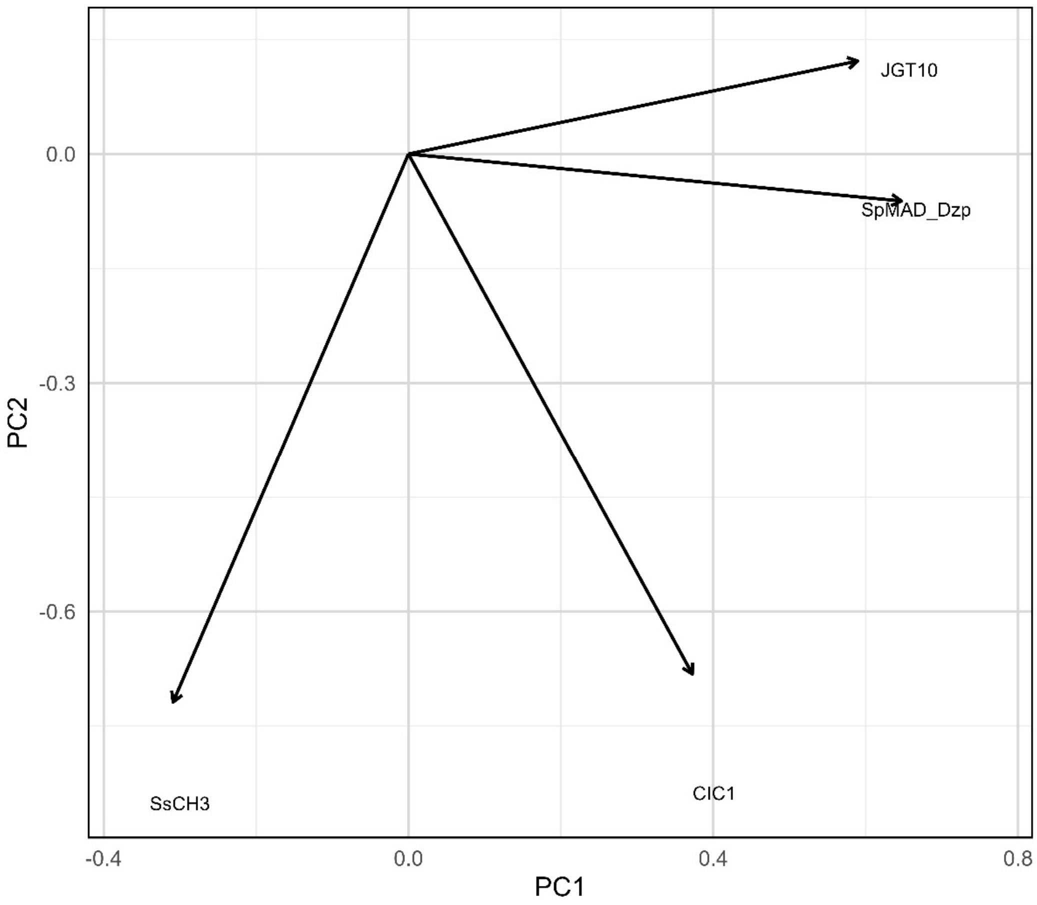

Figure S18. Loadings of the selected variables on the first two principal components (PC1 and PC2).

Additional analysis of response outliers:

The dataset includes 13 compounds belonging to the hydrocarbons class, and more than half of them are identified as response outliers. Compounds ID_RE 5, 6, 7, 10 and 12, based on their descriptor values, are positioned in the PCA space (Figure S16-S17) close to substances characterized by higher experimental removal efficiency values and are therefore overestimated by the experimental Radiative Efficiency values and are therefore overestimated by the model. In contrast, compounds ID_RE 2 and 3 are underestimated.

Compounds ID_RE 14 and ID_RE 20, belonging to the oxygenated hydrocarbons class, show opposite behaviours: the former is overestimated, whereas the latter is underestimated. Compound ID_RE 392, belonging to the hydrofluorocarbons class, is also underestimated.

Compound ID_RE 463, classified as an unsaturated chlorofluorocarbon/hydrochlorofluorocarbon, exhibits a particularly low experimental removal efficiency (logRE = −2.15) compared to the class average (−0.80). However, its molecular descriptor values are consistent with those of neighboring compounds in PCA space and with its class of belonging; structurally, it also appears similar to the other class members. As a result, the model overestimates its removal efficiency.

Compound ID_RE 472, belonging to the bromocarbons, hydrobromocarbons, and halons class, presents an experimental value consistent with its class but is nonetheless overestimated by the model. Compound ID_RE 505, characterized by a standardized residual close to the cut-off (−2.57), is likewise overestimated based on its descriptor values.

Finally, compounds ID_RE 585 and ID_RE 586 exhibit the highest experimental removal efficiency values within their class (−0.719). Both display values of the JGT10 descriptor (0.27 and 0.26) that are markedly lower than the class average (0.77). Consequently, these compounds are underestimated by the model and are positioned in the negative region of PC1.

Compounds ID_RE 663 and ID_RE 664, classified as special compounds, belong respectively to the training set and the test set and also display anomalous behaviour, consistent with their peripheral location in descriptor space.

Full model

$$\text{LogRE} = -1.67\ (\pm 0.05) + 1.03\ (\pm 0.08) \times \text{JGT10} + 0.06\ (\pm 0.006) \times \text{SpMAD\_Dz} - 0.12\ (\pm 0.03) \times CIC1 - 0.08\ (\pm 0.02) \times SsCH3 \quad (4)$$

N°: 670 R^2: 0.87, MAE_{tr}: 0.10, Q^2_{loo}: 0.87, R^2_{Yscr}: 0.01

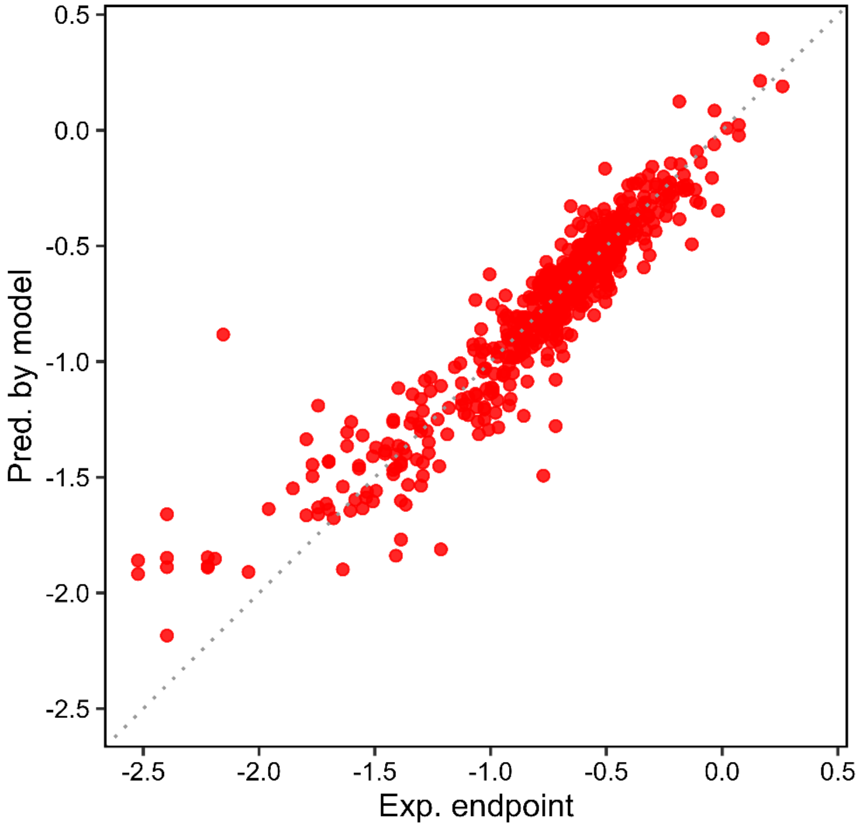

Figure S19. Regression diagnostic plots for the full MLR-QSAR model: comparison between experimental (Exp. endpoint) and model-predicted (Pred. by model) values for RE endpoint.

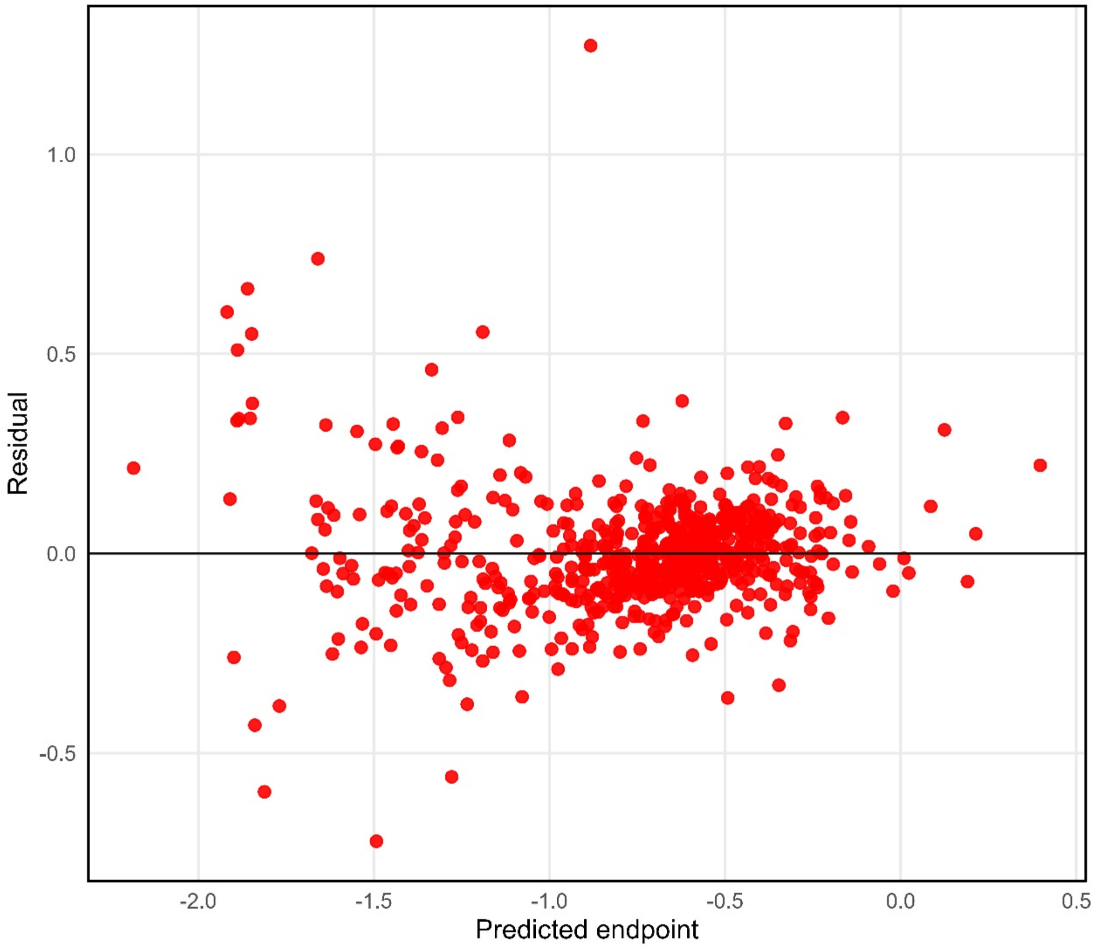

Figure S20. Plot of the residuals.

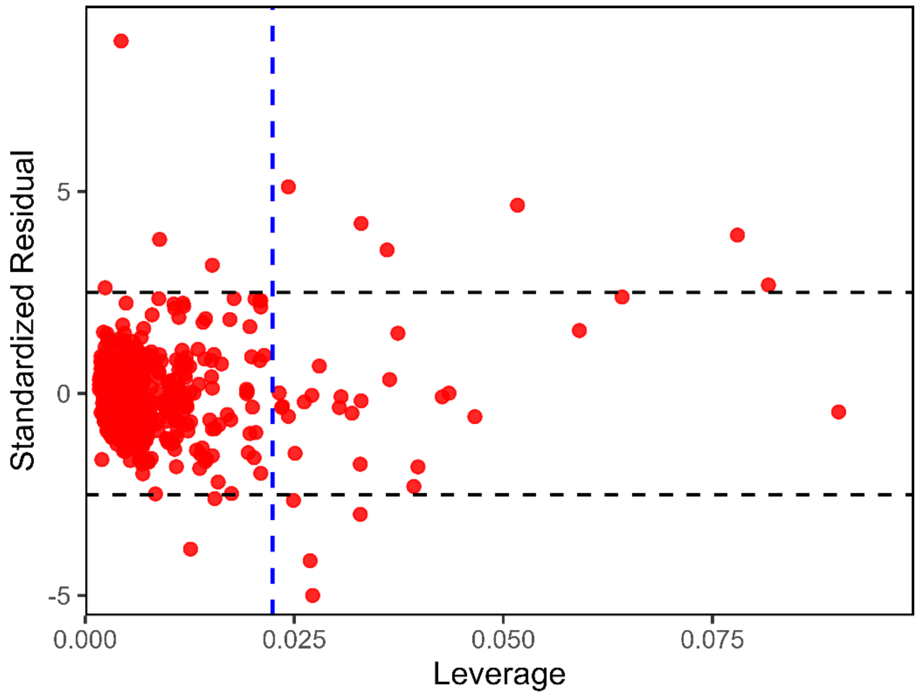

Figure S21. Williams plot of the full MLR-QSAR model of the RE endpoint, showing standardized residuals as a function of leverage. The horizontal dashed lines indicate the ±2.5 standardized residual thresholds used to identify response outliers,

while the vertical blue dashed line represents the leverage threshold (h= 0.0224), defining the applicability domain of the model.*

Standardized coefficients

Table S9. Standardized coefficient of the RE model equations obtained for the split and full datasets.

RE Model	JGT10	SpMAD_Dzp	CIC1	SsCH3
Split model	0.4924	0.4918	-0.1787	-0.173
Full model	0.4943	0.489	-0.1671	-0.1698

References

[1] Z. Yang, S. Yang, X. Wang, J. Xiao, e H. Wang, «Prediction of Global Warming Potential for Gases Based on Group Contribution Method and Chemical Activity Descriptor», *ACS Omega*, vol. 10, fasc. 22, pp. 22508–22520, giu. 2025, doi: 10.1021/acsomega.4c09710.

[2] S. Devotta, A. Chelani, e A. Vonsild, «Prediction of global warming potentials of refrigerants and related compounds from their molecular structure – An artificial neural network with group contribution method», *Int. J. Refrig.*, vol. 131, pp. 756–765, nov. 2021, doi: 10.1016/j.ijrefrig.2021.08.011.

[3] N. Rajapriya e K. Kawajiri, «Deep Learning for GWP Prediction: A Framework Using PCA, Quantile Transformation, and Ensemble Modeling», 28 novembre 2024, *arXiv*: arXiv:2411.19124. doi: 10.48550/arXiv.2411.19124.

[4] P. Blowers, K. F. Tetrault, e Y. Trujillo-Morehead, «Global warming potential predictions for hydrofluoroethers with two carbon atoms», *Theor. Chem. Acc.*, vol. 119, fasc. 4, pp. 369–381, mar. 2008, doi: 10.1007/s00214-007-0394-3.

[5] A. Kazakov, M. O. McLinden, e M. Frenkel, «Computational Design of New Refrigerant Fluids Based on Environmental, Safety, and Thermodynamic Characteristics», *Ind. Eng. Chem. Res.*, p. 120917100332001, set. 2012, doi: 10.1021/ie3016126.

[6] «Radiative forcing of climate by hydrochlorofluorocarbons and hydrofluorocarbons - Pinnock - 1995 - Journal of Geophysical Research: Atmospheres - Wiley Online Library». Consultato: 10 febbraio 2026. [Online]. Disponibile su: https://agupubs.onlinelibrary.wiley.com/doi/abs/10.1029/95JD02323

[7] X. Zhang, N. Kobayashi, M. He, e J. Wang, «Radiative Efficiency Estimation of Organic Substance Based on Group Contribution Method», *Energy Procedia*, vol. 61, pp. 492–495, 2014, doi: 10.1016/j.egypro.2014.11.1156.

[8] D. Betowski, C. Bevington, e T. C. Allison, «Estimation of Radiative Efficiency of Chemicals with Potentially Significant Global Warming Potential», *Environ. Sci. Technol.*, vol. 50, fasc. 2, pp. 790–797, gen. 2016, doi: 10.1021/acs.est.5b04154.

[9] Ø. Hodnebrog *et al.*, «Global warming potentials and radiative efficiencies of halocarbons and related compounds: A comprehensive review», *Rev. Geophys.*, vol. 51, fasc. 2, pp. 300–378, 2013, doi: 10.1002/rog.20013.

[10] G. Zhao, H. Kim, C. Yang, e Y. G. Chung, «Leveraging Machine Learning To Predict the Atmospheric Lifetime and the Global Warming Potential of SF_6 Replacement Gases», *J. Phys. Chem. A*, vol. 128, fasc. 12, pp. 2399–2408, mar. 2024, doi: 10.1021/acs.jpca.3c07339.

[11] R. Song, A. A. Keller, e S. Suh, «Rapid Life-Cycle Impact Screening Using Artificial Neural Networks», *Environ. Sci. Technol.*, vol. 51, fasc. 18, pp. 10777–10785, set. 2017, doi: 10.1021/acs.est.7b02862.

[12] Y. Geng *et al.*, «Machine learning-based prediction of global warming and ozone depletion potentials of refrigerants», *Chem. Eng. Sci.*, vol. 320, p. 122581, gen. 2026, doi: 10.1016/j.ces.2025.122581.
[13] R. Todeschini, D. Ballabio, e F. Grisoni, «Beware of Unreliable Q^2 ! A Comparative Study of Regression Metrics for Predictivity Assessment of QSAR Models», *J. Chem. Inf. Model.*, vol. 56, fasc. 10, pp. 1905–1913, ott. 2016, doi: 10.1021/acs.jcim.6b00277.
[14] R. Todeschini, *Introduzione alla chemiometria*. 1998.
